# Palliative care interventions and outcomes in patients with heart failure: an umbrella review

**DOI:** 10.1007/s10741-026-10632-4

**Published:** 2026-04-23

**Authors:** Jane Kim, Amanda Datesman, Hunter Groninger, Silas Selorm Daniels-Donkor, Santiago Bedoya Moreno, Anirudh Rao, Kelley Anderson

**Affiliations:** 1 University of Virginia, Charlottesville, VA USA; 2 MedStar, Washington Hospital Center, Washington, DC USA; 3 Georgetown University School of Medicine, Washington, DC USA; 4 INOVA Fairfax Medical Campus, Falls Church, VA USA

**Keywords:** Heart failure, Palliative care, Interventions, Patient-centered outcomes, Healthcare utilization

## Abstract

An expanding research base of systematic reviews has evaluated the effect of palliative care (PC) interventions delivered by primary or specialty PC providers for patients with heart failure (HF) and their caregivers. However, the overall evidence base has not been systematically examined. This umbrella review synthesizes the evidence regarding PC interventions among patients with HF to: 1) Describe interventions based on the palliative care domains in the AHA/ACC/HFSA HF guidelines, 2) Examine the effect of interventions on patient-centered and healthcare utilization outcomes, and 3) Provide recommendations for practice, policy and future research. Using the Joanna Briggs Institute methodology for umbrella reviews, we comprehensively searched for systematic reviews in MEDLINE, CINAHL, and Embase databases with no date restrictions. Published, peer-reviewed reviews were included. The search identified 19 reviews (high-quality, 11 with meta-analyses), of 104 unique primary studies after de-duplication (low to moderate quality). Primary and specialty PC was associated with improvements in quality of life, depression, advanced care planning, and reduced hospitalizations. The effect of PC on reducing symptom burden was inconclusive. Limited evidence demonstrated improvements in anxiety, satisfaction with care, caregiver burden, and hospital length of stay. PC did not significantly impact mortality rates or hospice referral. The concordance of the overall evidence was high, and although the quality of primary studies was overall low to moderate, findings confirm that primary and specialty PC offer meaningful benefits for patients with HF. Future research is required on caregiver outcomes, medically complex illness trajectories, intervention timing and fidelity, standardized outcome measurements, and longitudinal study designs with larger sample sizes.

**Supplementary Information:**

The online version contains supplementary material available at 10.1007/s10741-026-10632-4.

## Introduction/background

Heart failure (HF) remains one of the leading causes of morbidity and mortality worldwide, with an estimated prevalence of 67 million people with HF globally [1, 2]. Approximately 6.7 million Americans are diagnosed, and the lifetime risk of developing HF has risen to 1 in 4 adults. The management of HF is complex, with guideline recommendations encompassing primary prevention, optimization of guideline-directed medical therapy, patient education and self-management, rehabilitation/exercise therapy, implantable devices, and advanced surgical therapies [3]. Even with contemporary management, HF continues to be characterized by high healthcare costs, significant symptom burden, poor quality of life (QOL), prognostic uncertainty, and frequent hospitalizations [4–6]. To address the persistent challenges in HF care, a holistic and multidisciplinary approach is required to comprehensively support patients and their caregivers, including the integration of palliative care (PC).

PC focuses on relieving suffering, enhancing QOL, and prioritizes patient and caregiver-centered decision making by delivering comprehensive physical and emotional support. The 2022 American Heart Association/American College of Cardiology/Heart Failure Society of America (AHA/ACC/HFSA) HF guidelines provide a class 1 (level of evidence C) recommendation that palliative and supportive care, including high-quality communication, conveyance of prognosis, clarifying goals of care, shared decision-making, symptom management, and caregiver support, be provided to all patients with HF [3, 7].

A growing number of systematic reviews and meta-analyses have evaluated the effect of PC interventions delivered by primary (e.g. HF specialist, primary care) or specialty PC (specialized palliative care) providers on various patient-centered outcomes and healthcare utilization across a variety of care settings. Evidence of the benefits of PC in HF care is evolving; however, the overall evidence base of the effect of PC interventions on HF patient outcomes and care utilization has not been systematically examined. The proliferation of reviews has led to an incomplete evidence base with potential overlaps and conflicting conclusions.

Therefore, the purpose of this umbrella review is to comprehensively examine and integrate the evidence on the effect of PC interventions on patient-centered outcomes and resource utilization among patients with HF to synthesize these findings, appraise the collective quality of evidence, and provide clear, high-level guidance for practice, policy, and research. Specifically, our review is guided by the question: In patients with HF, what PC interventions (organized by the domains of care from the AHA/ACC/HFSA guidelines) are the most effective in improving patient and family-centered outcomes and resource utilization? We aim to: 1) Describe PC interventions based on the AHA/ACC/HFSA PC domains, 2) Analyze the effect of PC interventions on PC-related outcomes, including QOL, symptom burden, psychological status (depression or anxiety), hospitalization rate, hospital length of stay (LOS), mortality, healthcare utilization, hospice referral, advanced care planning (ACP), satisfaction with care, and caregiver burden, and 3) Provide recommendations for practice, policy and future research.

## Methods

### Umbrella review description and methods

An a priori protocol was developed in accordance with the Joanna Briggs Institute (JBI) Manual for Evidence Synthesis methodology on umbrella reviews. This protocol is registered in PROSPERO (CRD420251062735) and follows the JBI methodology for umbrella reviews [8, 9]. A JBI umbrella review is a systematic review of existing reviews and meta-analyses, designed to provide an overall examination of the body of evidence available for a given topic, assess the concordance of results, and highlight discrepant findings [9]. Preferred Reporting Items for Systematic Reviews and Meta-analyses (PRISMA) reporting guidelines were followed in reporting this review [10].

### Literature search

A database search strategy was developed and executed by a health sciences librarian. First, relevant keywords and controlled vocabulary terms were identified. An initial search of MEDLINE (PubMed), Embase (Elsevier), and PROSPERO determined that there were no currently published or registered umbrella reviews on this topic and identified relevant citations to inform the search strategy. Titles and abstracts of relevant reviews were examined for additional keywords and index terms to inform development of a comprehensive search strategy (Table SI).

The MEDLINE (PubMed), CINAHL (EBSCO), and Embase (Elsevier) databases were initially searched on 05/30/2025. The search was last updated on 10/17/2025. Results were limited to articles in English or Spanish, as translation services outside of the review team were not available. Due to the nature of the umbrella review, only published, peer-reviewed review articles were sought. Database-specific filters used in CINAHL and Embase to limit the results to peer-reviewed, published literature are noted alongside the search strategy in Table S1. No date limit was utilized for the search.

### Eligibility

Systematic reviews of studies that assessed the outcomes of PC interventions for adult (≥ 18 years) patients with HF were included. Participants in all care settings were considered, including but not limited to home/residential, PC units/hospices, long-term care facilities, acute care settings, and community clinic-based care. We included quantitative or mixed-methods systematic reviews with or without meta-analyses. Narrative reviews, scoping reviews, rapid reviews, and other nonsystematic reviews, as well as primary research studies, were excluded. Only systematic reviews published in English and Spanish were considered. No date limitations were applied. Only published systematic reviews were considered.

Eligible reviews needed to include a description of the review question, eligibility criteria, a clear and comprehensive search strategy in at least two databases, and critical appraisal by at least one reviewer and confirmed by another or discussed among the team, using a standardized tool.

### Screening and data extraction

Two reviewers (KMA and JK) screened all retrieved citations for eligibility. Reviewers worked independently in both the title/abstract and full-text screening stages. Conflicts between reviewers were resolved by consensus. After the initial two-stage screening process, reference lists of included papers were examined for further relevant reviews, but no additional relevant records were identified. We reported the screening process using the PRISMA flow diagram. Data were extracted from each included review using the JBI data extraction tool for Systematic Reviews and Research Syntheses [9] detailing the systematic review’s author, year, objective, participant characteristics, setting, interventions, databases searched, date range of included studies, number of studies, type and country of origin of included studies, appraisal rating, type of review, outcomes, and results (Table SII).

### Data summary

We used the Graphical Representation of Overlap for OVErviews (GROOVE) tool to calculate double counting of primary studies in the included reviews. We appraised overlap according to the percentage of corrected covered area (CCA): 0% − 5% (slight overlap), 6% − 10% (moderate overlap), 11% − 15% (high overlap), and above 15% (very high overlap) [11, 12]. We narratively synthesized the findings and created summary tables to present key characteristics of included reviews, descriptions of PC interventions based on HF guideline domains, and patient-centered and healthcare utilization outcomes following PC interventions. We prioritized the most recent, comprehensive, or high-quality systematic reviewers and/or meta-analyses in our results synthesis; otherwise narrative.

## Results

### Review selection

**Fig. 1:**
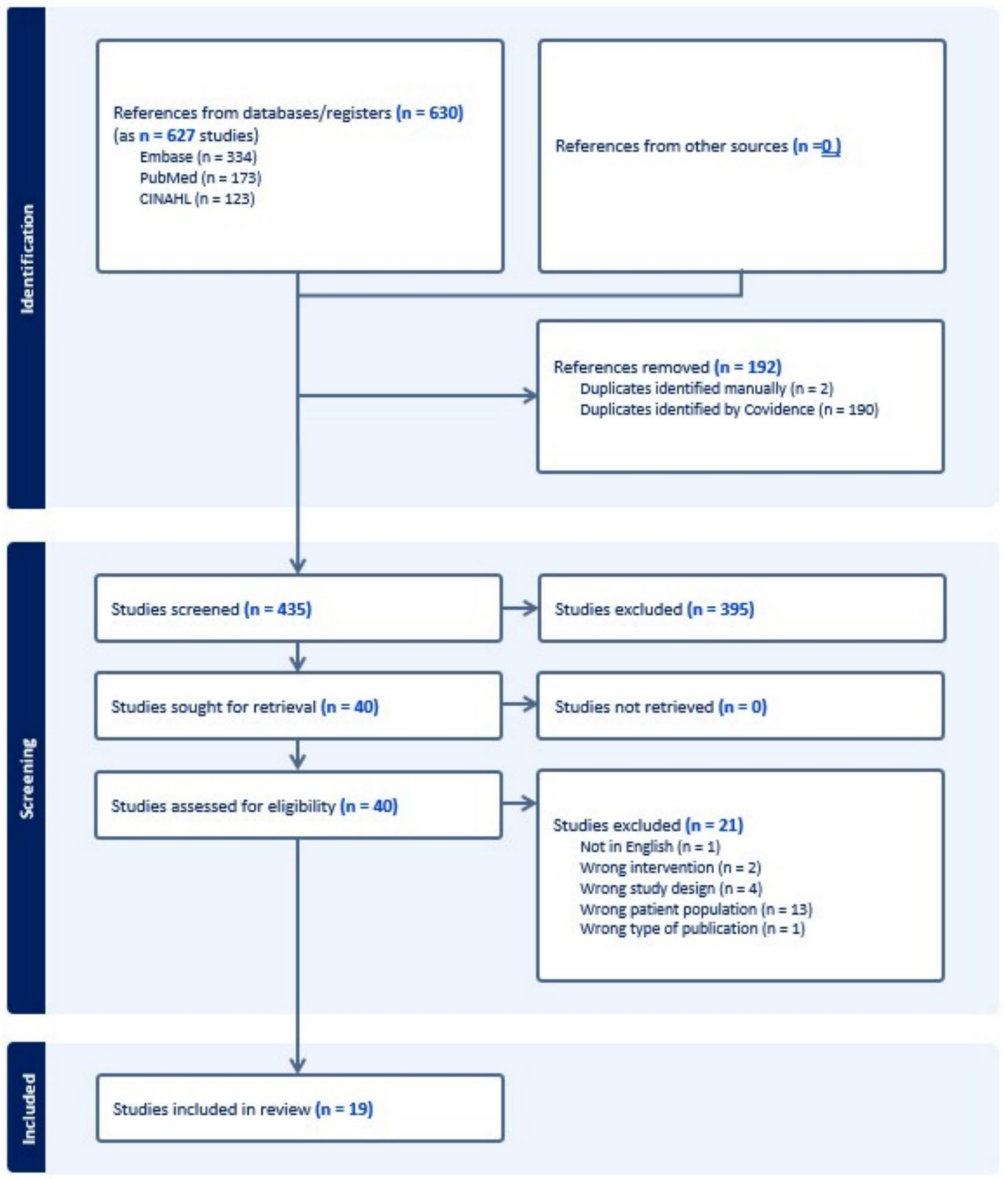
PRISMA flow diagram. Study selection process

After searches in the electronic databases, 630 citations were identified and imported into Covidence review management software (Fig. 1). Covidence identified 190 duplicate records. The review team identified two more duplicate records as well as three records that reported on the same study as another record in the review. The team screened the remaining 435 citations against the pre-defined inclusion/exclusion criteria in a blinded, dual-screener title and abstract screen. Of the 435 records, 40 were considered in a dual-screener full-text review stage. Two reviewers assessed the eligibility of integrative reviews during the review stage and determined that they met all pre-determined inclusion criteria and qualified as a systematic review by including a description of the review question, clear eligibility criteria, a comprehensive search strategy in at least two databases, and critical appraisal by at least one reviewer and confirmed by another or discussed among the team, using a standardized tool. 19 items were included in the final analysis.

### Methodological quality

All 19 reviews were independently appraised by two authors (SSD and SBM) using the JBI critical appraisal checklist for systematic reviews [8], resolving any disagreements through discussions and consensus. Each review was assessed using item-level yes/no/unclear criteria and depicted using color-coded icons in the quality appraisal summary table. The overall methodological quality was high (Table 1). All reviews were categorized as high quality (meeting ≥ 75% of criteria) according to categorizations adopted from previous studies [13, 14]. Each review distinctly specified its research question, utilized appropriate inclusion criteria, and implemented rigorous search methods with sufficient coverage of sources. Almost all reviews employed suitable criteria to assess the primary studies and synthesized evidence utilizing appropriate methodologies. Moreover, each review presented conclusions and recommendations substantiated by the data, providing at least some guidance for future research.

**Table 1:**
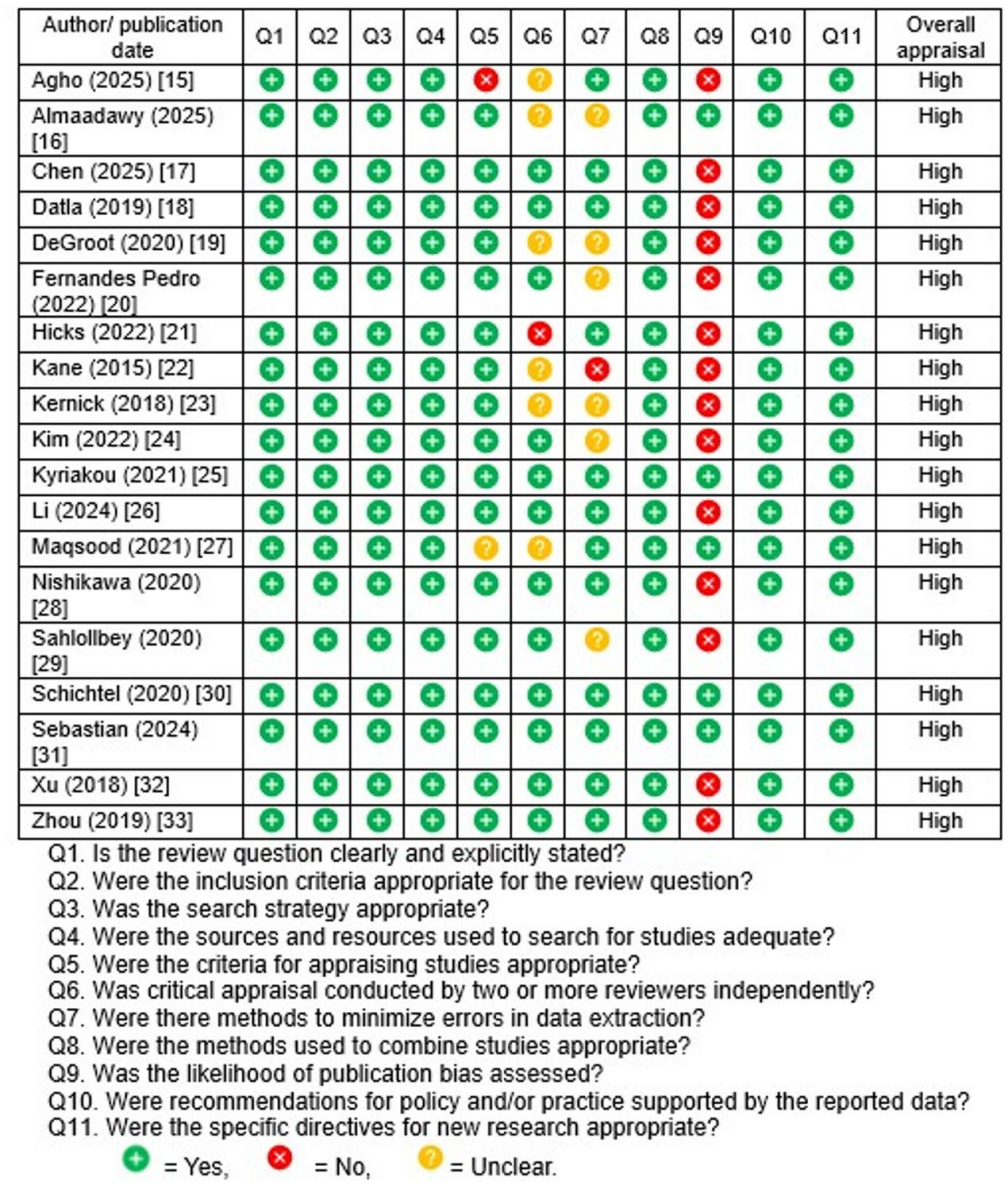
JBI critical appraisal of the included systematic reviews (*n* = 19)

However, other appraisal criteria were addressed with less consistency. Publication bias was addressed in only approximately one-third of the reviews, rendering it the most commonly unmet criterion across studies [16, 25, 27, 30, 31]. Similarly, approximately 35% of the reviews failed to explicitly indicate the involvement of two independent reviewers for critical appraisal or duplicate data extraction, resulting in “No” or “Unclear” ratings in those domains [15, 16, 19, 21–23, 27].

### Risk of bias and certainty of evidence

Among primary studies, there was overall high risk of performance bias due to lack of blinding of participants or personnel to the intervention groups due to the nature of PC interventions. There was high risk of detection bias for subjective outcomes (i.e. self-reported measures of QOL or symptoms) since blinding was not possible for these measurements. Some studies had high attrition rates, leading to bias in the intervention effect estimate (Supplementary Table II).

The Risk of Bias in Systematic Reviews (ROBIS) tool was used to assess the risk of bias in the included reviews [34]. Most reviews demonstrated low risk of bias in the study eligibility criteria, data collection, appraisal, and synthesis of findings domains. However, several reviews demonstrated high risk of bias in their selection of studies, due to a single author performing title and abstract screening (Supplementary Table III).

We used the Grading of Recommendations Assessment and Evaluation (GRADE) approach modified for umbrella reviews to assess the certainty of evidence and arrive at an overall certainty judgment for each outcome. Following guidance by Dullea et al. [35], a modified GRADE approach for complex reviews addresses both the primary study risk of bias as assessed by the included reviews in addition to the risk of bias of the systematic reviews. Our decisions to downgrade the quality of evidence for each outcome are explained in the footnotes of the modified GRADE table (Supplementary Table IV). We judged the quality of the evidence to be moderate for anxiety, hospitalization, and mortality outcomes. We judged the quality of evidence as low for QOL, depression, hospice referral, ACP, and very low for symptoms, hospital LOS, ED visits, satisfaction with care, and caregiver burden.

### Study overlap

**Fig. 2:**
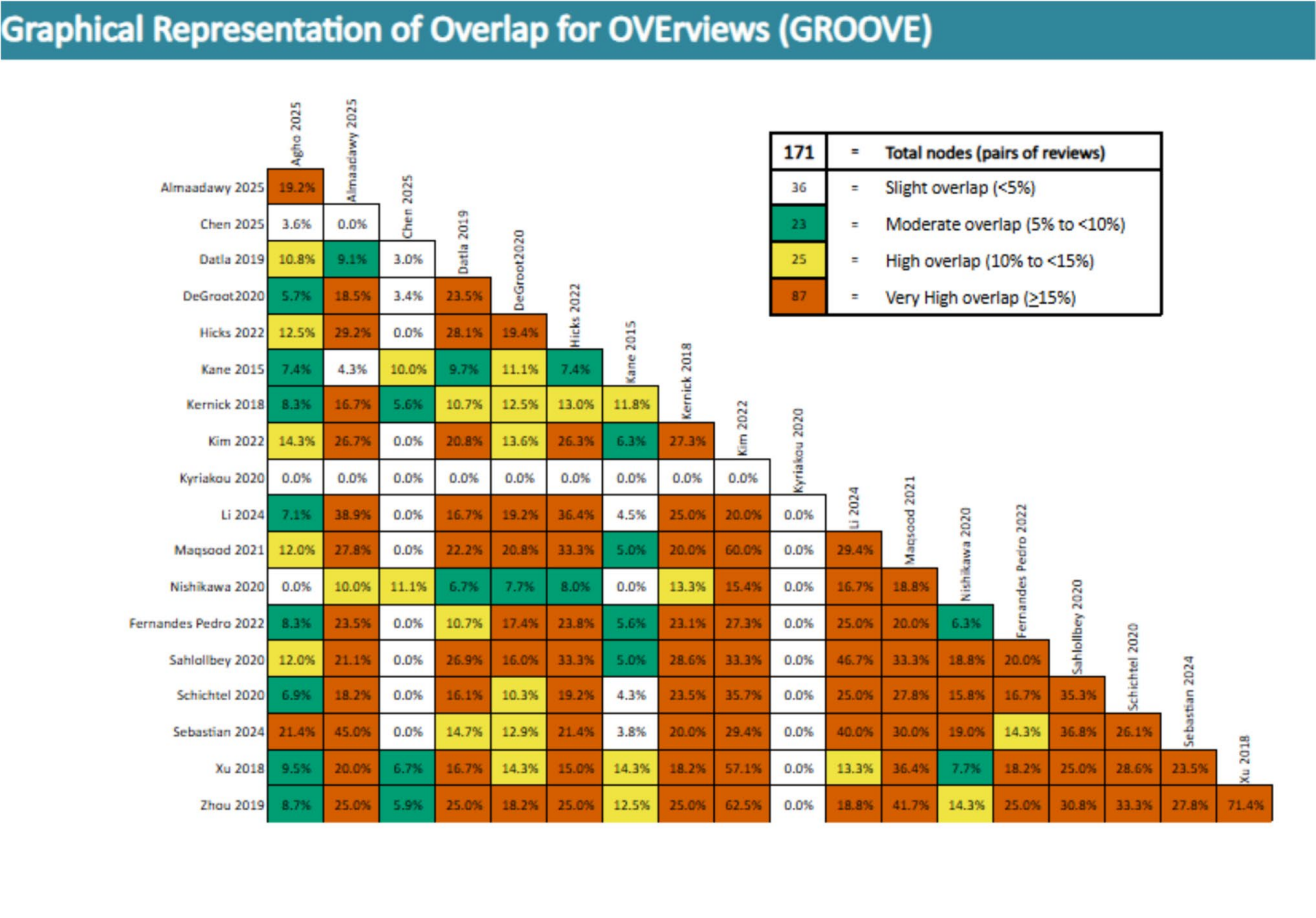
Graphic Representation of Overlap OVErviews (GROOVE). The GROOVE tool calculates double counting of primary studies in the included reviews. The percentage of corrected covered area (CCA) demonstrated a moderate degree of overlap (6.3%) between primary studies

Graphical Representation of Overlap for OVErviews (GROOVE) tool was used to calculate double counting of primary studies in the included reviews [12]. The 19 systematic reviews covered 104 unique primary studies after de-duplication. Studies were counted as unique based on nonoverlapping citations in the GROOVE matrix. Overall, the CCA demonstrated a moderate degree of overlap (6.3%) between primary studies (Fig. 2). Overlap between reviews ranged 0.0% − 71.4%. The highest degree of overlap (71.4%) was between the five studies in the Xu review [32] which were all included in the seven studies in the Zhou review [33].

### Characteristics of systematic reviews

The 19 reviews were published from 2015 to 2025 (Table 2). Primary studies were conducted in North America (62 studies), followed by Europe (25 studies), Asia (10 studies), Eurasia (5 studies), South America (1 study), and Oceania (1 study). Most studies originated from the United States (57 studies) and the United Kingdom (7 studies). 15 reviews were quantitative [16, 18, 20, 24–35] (10 with meta-analyses) [16, 25–33], and 4 were mixed methods [15, 17, 19, 21] (1 with meta-analysis) [17]. The total number of participants evaluated in the reviews ranged from 545 [32] to 19,891 [18]. All reviews focused on adult patients 18 years or older with HF, predominantly with NYHA class II to IV, including both male and female sexes.

**Table 2:** Characteristics of included systematic reviews

Authors, year, country	Study design	Primary studies included in review	Sample	Settings	Type of intervention	Outcomes and measures	Results
Agho et al. 2025 [15] USA	Mixed-methods Systematic review and narrative synthesis	18 (RCT, cohort, observational, clinical practice guideline, implementation study, qualitative)	7604 adults with HF	Settings not reported	Primary and specialty PC	QOL Measures not reported	PC is effective in improving QOL among patients with HF
Almaadawy et al. 2025 [16] USA	Systematic review and meta-analysis	13 RCT	1541 adults with HF	Hospital home, telehealth, telephone	PC intervention (primary or specialty PC not specified)	-QOL (KCCQ, ESAS, ED5Q, FACIT-Pal, PHQ-9, MLHFQ) -Mortality -Symptoms (GSDS, Borg Scale) -Anxiety (GAD-7) -Depression (PHQ-9, PHQ-4, HADS)	PC significantly improved QOL and reduced anxiety No significant differences were observed for dyspnea, depression, or mortality
Chen et al. 2025 [17] China	Mixed-methods systematic review and meta-analysis	11 (RCT, quasi-experimental, mixed method, qualitative)	2809 adults ages 50 + with HF	Hospital	ACP	-ACP completion Measures not reported	Three studies showed ACP-related completion after ACP intervention
Datla et al. 2019 [18] UK	Systematic review and narrative synthesis	23 (RCT, pilot study, quasi-experimental, cohort, case–control, cross-sectional)	19891adults with NYHA class III-IV HF	Community, hospital, hospice	Primary or specialty PC intervention apart from ACP alone	-Symptoms (ESAS, MSAS) -Depression (PHQ-9, HADS, QIDS) -QOL (KCCQ, EQ5D) -Mortality -Rehospitalization	PC interventions showed statistically significant benefits for symptom burden, depression, QOL, reducing rehospitalizations and hospital LOS No significant difference were observed for mortality or hospice use
DeGroot et al. 2020 [19] USA	Mixed-methods Systematic Review and narrative synthesis	19 (RCT, prospective, descriptive, chart review, qualitative, mixed-methods)	1231 adults with HF, informal caregivers or HF providers	Home, community, hospital, telephone	Primary or specialty PC	-Symptoms (ESAS) -QOL (KCCQ, MQOL) -Depression -Anxiety -Rehospitalization -ACP	Outpatient PC improved QOL, depression, alleviated symptoms, and decreased rehospitalization
Fernandes Pedro and Reis-Pina 2022 [20] Portugal	Systematic review and narrative synthesis	7 (RCT, cohort, prospective)	5388 participants with NYHA class II-IV HF	Settings not reported	Primary or specialty PC	-QOL -Symptoms -Hospital readmission Measures not reported	PC was associated with significant improvements in QOL and symptoms Mixed evidence showing improvements in hospital readmission
Hicks et al. 2022 [21] UK	Mixed-methods systematic review and narrative synthesis	18 (RCT, mixed-methods)	1817 adults with NYHA class II-IV HF	Community, outpatient, hospital, mixed settings	Primary or specialty PC	-QOL (CHQ-C, EQ5D, FACIT-PAL, KCCQ, MLHFQ) -Symptoms (GSDS) -psychological status (BDI, GAD-7, HADS, PHQ-9) -Satisfaction with care -Mortality -ACP -Rehospitalization -Caregiver burden (ZBI)	PC interventions showed benefit for QOL, psychological status, satisfaction with care, symptom burden, reductions in hospitalization, and caregiver burden No significant differences were found for mortality or ACP
Kane et al. 2015 [22] UK	Systematic review and narrative synthesis	10 (RCT, feasibility, prospective, retrospective)	2540 adults with NYHA class II-IV HF	Hospital, community	Primary or specialty PC	-QOL (KCCQ, MLHFQ) -Symptoms (ESAS) -Depression (PHQ-9)	PC reduced symptom burden, depression, and improved QOL
Kernick et al. 2018 [23] UK	Systematic review and narrative synthesis	8 (RCT, observational)	14357 participants with HF	Community, hospital	Primary or specialty PC	-Hospital admissions -QOL (MQOL, KCCQ, FACIT-Pal) -Symptoms (ESAS, CHQ-Chinese, MQOL-Chinese)	ACP interventions led to statistically significant reductions in hospital readmission and increased use of palliative services
Kim et al. 2022 [24] South Korea	Integrative review	6 RCT	Adults with HF (sample size not reported)	Hospital, home, telephone	Primary or specialty PC	-QOL (KCCQ, MLHFQ, EQ5D, MQOL, CHFQ, FACIT-Pal) -Symptom burden (ESAS) -Depression (HADS) -Hospital readmission -ACP documentation -Mortality -Hospice use	Most studies showed statistically significant improvements in QOL, depression and symptom burden following PC intervention No significant differences between groups in hospice use
Kyriakou et al. 2020 [25] Cyprus	Systematic review and meta-analysis	10 RCT	3144 adults with HF	Community, home	Supportive care interventions (communication, education, psychosocial/spiritual, or symptom management)	-QOL (MLHFQ, SF-36, KCCQ, Quality of Life Index) -Depression (HADS, BDI) -Anxiety (HADS)	Overall effect indicated a positive effect of supportive care on QOL Supportive care was found to have a nonsignificant effect on depression
Li et al. 2024 [26] China	Systematic review and meta-analysis	11 RCT	1535 community-dwelling adults with HF	Hybrid (face-to-face and telehealth), home, hospital	Primary or specialty PC	-QOL (SF-36, FACIT-Pal, KCCQ, EQ5D, QOL Index, MLHFQ, MQOL, CHFQ) -Symptoms (ESAS, HF Symptom Survey) -Anxiety (GAD, HADS, Kessler Scale) -Depression (HADS, PHQ-9) -Mortality	PC interventions demonstrated statistically significant effects on improving health-related QOL and HF-specific QOL, and reduced anxiety and depression PC interventions did not affect mortality or symptom burden
Maqsood et al. 2021 [27] USA	Systematic review and meta-analysis	10 RCT	4057 adults with HF	Hospital and emergency department	PC intervention (type of PC not specified)	-QOL -Hospitalization -ACP -Symptoms Measures not reported	PC led to a significant reduction in hospital admissions and increased ACP No significant differences were observed for QOL, change in ED visits, or symptoms
Nishikawa et al. 2020 [28] Japan	Systematic review and meta-analysis	9 RCT	1242 adults with HF	Hospital, community	ACP interventions	-QOL (KCCQ, MLHFQ, EQ5D, FACIT-Pal) -Depression (PHQ-8, PHQ-9, HADS) -Hospice use -ACP -Mortality	ACP interventions did not show improvements in QOL ACP improved depression All-cause mortality might have increased in participants who received ACP intervention
Sahlollbey et al. 2020 [29] Canada	Systematic review and meta-analysis	10 RCT	1050 adults (87.7% with HF)	Hospital, outpatient, mixed	Primary and specialty PC	-QOL -Symptom burden -Hospitalizations -Mortality Measures not reported	PC interventions were associated with a significant reduction in hospitalization, modest improvements in QOL, and modest reduction in symptom burden There was no significant difference regarding mortality
Schichtel et al. 2020 [30] UK	Systematic review and meta-analysis	14 RCT	2924 adults with HF	Community, hospital	Primary and specialty PC, focus on ACP	-QOL -Satisfaction with care Measures not reported	ACP was associated with a statistically significant improvement in QOL and patient satisfaction
Sebastian et al. 2024 [31] India	Systematic review and meta-analysis	16 RCT	2324 adults with HF	Home, hospital	Primary or specialty PC or palliative telehealth interventions	-QOL (KCCQ, FACIT-Pal) -Hospitalization -Mortality	Patients who received PC or palliative care telehealth interventions experienced improved QOL and a decrease in hospitalizations There was no significant change in all-cause mortality
Xu et al. 2018 [32] China	Systematic review and meta-analysis	5 RCT	545 adults with HF	Settings not reported	PC interventions (primary or specialty PC not specified)	-Hospital readmission -QOL (MLHFQ) -Symptoms (ESAS) -Depression (PHQ-9) -Mortality	PC intervention was associated with a significantly decreased readmission, symptoms, and depression PC was not associated with decreased mortality and did not influence QOL
Zhou and Mao 2019 [33] China	Systematic review and meta-analysis	7 RCT	769 adults with HF	Settings not reported	PC interventions (primary or specialty PC not specified)	-QOL -Depression -Mortality -Rehospitalization Measures not reported	PC was associated with significantly increased QOL and reduced depression scores PC demonstrated no impact on mortality or rehospitalization

*AD* Advanced directive; *ACP* Advanced care planning; *BDI* Beck’s depression inventory; *CHFQ* Chronic heart failure questionnaire; *CHQ-C* Chronic heart failure questionnaire- Chinese; *ESAS* Edmonton symptom assessment scale; *EQ5D* EuroQol-5D; *FACIT-PaL* Functional assessment of chronic illness therapy- palliative care scale; *GAD-7* Generalized anxiety disorder questionnaire; *GSDS* General symptom distress scale; *HADS* Hospital anxiety and depression survey; *HF* Heart failure; *HRQOL-14* Health-related quality of life scale; *KCCQ* Kansas City cardiomyopathy questionnaire; *LOS* Length of stay; *NYHA MLHFQ*, Minnesota living with heart failure questionnaire; *MSAS* Memorial symptom assessment scale; *MQOL* McGill quality of life questionnaire; *NYHA* New York heart association; *PC* Palliative care; *PHQ-4/PHQ-9* Patient health questionnaire, *QIDS* Quick inventory of depressive symptomology; *QOL* quality of life; *RCT* Randomized controlled trial; *SF-36* 36-Item short form survey; *ZBI* Zarit Burden interview instrument

18 reviews included studies evaluating PC interventions across a mixture of settings (hospital, home, hospice, community, telehealth, hybrid, telephone) while one review evaluated PC interventions within the hospital setting only [17]. Most reviews included both primary and specialty PC interventions. All reviews provided data on at least one of our PC intervention domains and outcomes of interest. QOL was evaluated most often (17 reviews) [15, 16, 18–22, 24–33], followed by symptoms (11 reviews) [16, 18–22, 24, 26, 27, 29, 32], while hospital LOS [18] and caregiver burden [21] were evaluated in a single review, respectively.

### Description of PC interventions

A total of 81 PC interventions were evaluated across the included reviews. Interventions included both primary and specialty PC providers (with specialty PC comprising the majority) across multiple disciplines, including physicians, nurses and nurse specialists, physiotherapists, psychiatrists, pharmacists, dieticians, social workers, and chaplains. The AHA/ACC/HFSA HF guidelines detail six palliative and supportive care domains to improve care processes and patient outcomes which add to overall HF management. These include: high-quality communication, conveyance of prognosis, clarifying goals of care, shared decision making, symptom management, and caregiver support [3]. Most PC interventions were multi-component, addressing multiple domains of PC in an integrated manner (Table 3). Among the interventions, high quality communication was most often incorporated, followed by goals of care clarification, symptom management, shared decision-making, conveyance of prognosis, and lastly, caregiver support. Thematic descriptions of PC interventions grouped by the HFSA guideline PC domains are described below.

**Table 3:** Description of palliative care interventions

AHA/ACC/HFSA Palliative Care Domains	Description of Interventions
High-quality communication	• Interdisciplinary care team members provided face-to-face or remote counseling services • Discussions facilitated problem-solving, complex decision-making, patient education, HF self-management, and psychosocial support
Conveyance of prognosis	• Assessments evaluated the patient’s prognosis and understanding of illness • Discussions facilitated preparation for end-of-life
Clarifying goals of care	• Person-centered approaches explored patient’s goals, values, and beliefs • Focused on patient preferences for treatment, place of care, and type of care (life-prolonging, limited medical care, or comfort care)
Shared decision making	• Conversations engaged patients, family members or surrogates, and care team members • Focused on care plan development, appointing surrogates, discussing preferences, care coordination, and advanced care planning
Symptom management	• Facilitated the assessment, management, and alleviation of physical and psychological (depression, anxiety) symptoms
Caregiver support	• Caregivers received counseling and support from multidisciplinary care team members to strengthen home care skills, symptom monitoring, and HF management skills

#### High quality communication

High-quality communication was included as a component in 59 (72.8%) PC interventions across all the reviews. High-quality communication was conducted through face-to-face counseling services by providers or members of a multidisciplinary care team. Remote/telephonic communication was also utilized, particularly for follow-up communication. Frequent and high-quality communication facilitated problem-solving, complex decision-making, patient education, and HF self-management. Conversations included discussions on changes in patient status, risk factor management, emotional or psychosocial support, as well as targeted recommendations based on clinical data. Motivational interviewing was used in some interventions to promote positive behavior change among patients.

#### Clarifying goals of care

Interventions that focused on goals of care (GOC) were found among 46 (56.7%) interventions across all the reviews. Interventions incorporating GOC took a person-centered approach based on patient goals, values, and beliefs, and explored future decision-making, accounting for the patient’s rationale for GOC wishes. Some interventions utilized surveys on treatment preferences. Preference for place of care was also discussed. Conversations on different types of goals were conducted, including life-prolonging care, limited medical care, and comfort care.

#### Symptom management

Symptom management was a focus of 45 (55.5%) PC interventions across the reviews. Interventions focused on physical symptoms, including dyspnea, fatigue, nausea, pain, constipation, edema, and insomnia, as well as psychological symptoms, including depression and anxiety. Multidisciplinary team members assessed symptom burden, frequency, and severity. Nurses provided education on HF symptoms and strategies for self-care to assist patients in monitoring, interpreting, and managing their symptoms. Both pharmacologic and nonpharmacologic measures were utilized to facilitate symptom relief. Interventions addressing depression incorporated mental health assessments, depression education, and coaching on antidepressant management.

#### Shared decision making

Shared decision making (SDM) was incorporated in 32 (39.5%) interventions across the reviews. SDM was often conducted between the patient, a surrogate or family member, and a care team member. Conversations focused on developing the care plan, appointing surrogates, discussing preferences, care coordination, and advance care planning.

#### Conveyance of prognosis

Conveyance of prognosis was included as a component in 24 (29.6%) interventions across all the reviews. Interventions focusing on the conveyance of prognosis were often conducted in tandem with high-quality communication. Interventions assessed the patient’s prognosis/condition and evaluated the patient's understanding of their illness. Interventions facilitated patients’ preparation for end-of-life. Conversations about prognosis often involved family members or surrogates, with some interventions directly preparing surrogates to make future decisions to honor patient choices.

#### Caregiver support

Only 6 (7.4%) PC interventions addressed caregiver support across 9 reviews [15, 16, 18, 19, 21, 22, 25–27]. Caregivers received counseling and support from a dietician, psychotherapist, social worker, or occupational therapist. One intervention consisted of a telephone-based psychosocial intervention to address depression symptoms, and support to informal caregivers was provided as needed [36]. Another intervention featured telephone coaching to caregivers on HF home care skills, including symptom monitoring, medication adherence, and fluid restrictions. Materials were sent to caregivers by mail, including a caregiver's guide, a list of local support organizations, a pill organizer, and a book for HF caregivers [37].

### Description of PC related outcomes

Overall, there was good concordance across the reviews regarding our outcomes of interest following PC intervention (Table 4). Findings from the reviews showed that overall, primary and specialty PC interventions were effective in improving patient QOL, depression, ACP, and reducing hospitalization. Results were mixed regarding the effect of PC on symptoms. Few reviews examined the effect of PC interventions on anxiety, satisfaction with care, caregiver burden, and hospital LOS, but results favored PC intervention. Consistently across the reviews, no significant differences were found between PC intervention groups and usual care regarding mortality or hospice referral. Detailed descriptions of outcomes and instruments are provided below.

**Table 4:**
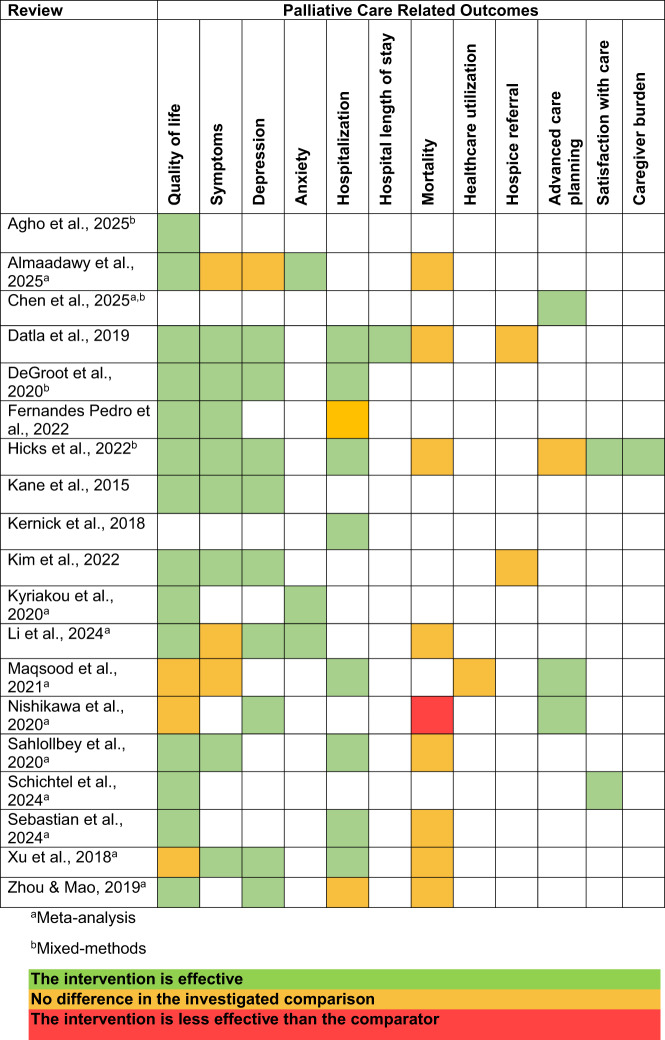
Outcomes related to palliative care interventions

#### Quality of life

QOL was evaluated in 17 reviews. 14 reviews found that PC intervention was effective in improving QOL [15, 16, 18–22, 24–26, 29–31, 33], while 3 reviews found no difference between PC interventions compared to usual care [27, 28, 32]. Measures used to assess QOL most often included the Kansas City Cardiomyopathy Questionnaire (KCCQ), Functional Assessment of Chronic Illness Therapy-Palliative Care scale (FACIT-Pal), and Minnesota Living with Heart Failure Questionnaire (MLHFQ). Other measures for QOL included the Patient Health Questionnaire, EuroQol-5D (EQ5D), McGill QOL Questionnaire (MQOL), Chronic Heart Failure Questionnaire (CHFQ), 36-Item Short Form Survey (SF-36), Quality of Life Index, the Chronic HF Questionnaire, the Health-Related Quality of Life (HRQOL-14) Scale, and Chronic Heart Failure Questionnaire-Chinese (CHQ-C).

The highest quality of evidence of the effect of PC interventions on QOL were reported in several meta-analyses. Almaadawy et al.’s, meta-analysis of 9 studies found that PC significantly improved QOL scores compared with usual care (SMD 1.36, 95% CI 0.89 to 1.83, *p* < 0.00001, I^2^ = 0%), with notable improvements using the KCCQ [16]. Similarly, Li et al.’s meta-analysis of 6 studies showed a significant improvement in QOL favoring PC interventions (SMD 0.30, 95% CI 0.12 to 0.48, I^2^ = 0%) with moderate quality of evidence. The pooled SMD was converted into a clinically intuitive MD based on the KCCQ and was calculated at 6.31 (95% CI 2.52 to 10.10) [26]. Kyriakou et al. found that PC intervention indicated a positive effect on QOL (SMD −9.44, 95% CI 15.54 to −3.33, *p* = 0.002) [25]. Sebastian et al. demonstrated a statistically significant improvement in QOL among patients who received PC or palliative telehealth interventions (WMD for KCCQ was 3.56, 95% CI 0.43 to 6.69, *p* = 0.03, *I* ^2^ = 46%) and for FACIT-Pal was 2.54 (95% CI 1.00 to 4.08, *p* = 0.001, *I*^2^ = 14%) [31]. However, Nishikawa et al. observed no difference in QOL between the ACP intervention group and usual care (SMD 0.06, 95% CI −0.26 to 0.38, *p* = 0.71; low-quality evidence) [28].

#### Symptoms

Symptoms were evaluated among 11 reviews, with 8 reviews reporting that PC intervention improved symptoms [18–22, 24, 29, 32], while 3 meta-analyses found no difference between PC and usual care groups [16, 26, 27]. Measures used to assess symptoms most commonly included the Edmonton Symptom Assessment Scale (ESAS). Other measures included the Borg Scale, General Symptom Distress Scale (GSDS), Memorial Symptom Assessment Scale, Chronic Heart Failure Questionnaire, and the HF Symptom Survey.

Sahlollbey et al. found that compared with usual care, PC interventions were associated with a modest reduction in symptom burden (SMD −0.29, 95% CI −0.54 to 0.03, *I*^2^ = 15%) [29]. Similarly, Xu et al. also described that PC intervention showed significantly reduced symptoms (SMD −2.5, 95% CI −4.39 to −0.62, *p* = 0.009) [32]. However, three other meta-analyses showed nonsignificant differences in symptom burden following PC intervention. Almaadawy et al. found that overall, there was no significant difference between PC and usual care groups in change from baseline in dyspnea scores (MD − 0.15, 95% CI − 0.66 to 0.36, *p* = 0.58). Results remained consistent between the overall and low-risk-of-bias analyses, showing consistent results regardless of the studies’ methodological rigor [16]. Likewise, Li et al. found that PC interventions did not significantly reduce symptom burden (SMD − 0.09, 95% CI − 0.40 to 0.21, *I*^2^ = 0%, low quality of evidence). The translated effect size on the ESAS was − 1.25, 95% CI −5.2 to 2.7) [26]. Maqsood et al. also noted a nonsignificant decrease in symptoms between PC and usual care groups (WMD −3.05, 95% CI −7.15 to 1.04) [27].

Depression was evaluated in 10 reviews. 8 out of 10 reviews found that PC significantly improved depression compared with usual care, with low to moderate quality of evidence [18, 21, 22, 24, 26, 28, 32, 33]. Measures used to evaluate depression included the Patient Health Questionnaire (PHQ-9), Hospital Anxiety and Depression Survey (HADS), Quick Inventory of Depressive Symptomatology, and Beck’s Depression Inventory (BDI).

Li et al. examined 6 studies and found PC reduced depression (SMD −0.18, 95% CI 0.33 to −0.03, *I*^2^ = 20%, low quality of evidence). The translated effect size on the HADS was − 0.77 (95% CI − 1.4 to − 0.13) [26]. Nishikawa et al. found ACP improved depression compared with usual care (SMD −0.58, *p* < 0.00001, low quality evidence) [28]. Xu et al. found PC intervention was associated with significantly reduced depression (SMD −1.16, 95% CI −1.73 to −0.58, *p* < 0.005) [32]. Zhou & Mao also found PC reduced depression scores (SMD −0.62, 95% CI −0.99 to −0.25, *p* = 0.03) [33]. However, Almaadawy et al. found no significant differences for depression between groups (MD − 0.34, 95% CI − 0.91 to 0.22, *p* = 0.23). After conducting a subgroup analysis based on the depression scale used, the pooled mean difference did not favor either of the 2 groups in both HADS and ESAS subgroups (HADS: MD − 0.28, 95% CI − 1.02 to 0.46, *p* = 0.45; ESAS: MD − 0.12, 95% CI − 1.76 to 1.52, *p* = 0.09) with homogenous studies within each subgroup [16]. Kyriakou et al. found that PC had a positive effect on depression, but results were not statistically significant (SMD −0.53, *p* = 0.13) [25].

Anxiety was evaluated in 3 reviews, with reviews demonstrating that PC intervention improved anxiety. Measures used to evaluate anxiety included the HADS, General Anxiety Disorder scale (GAD-7), and Kessler Scale. Almaadawy et al. found that PC intervention significantly reduced anxiety scores (MD −0.39, 95% CI −0.66 to −0.11, *p* = 0.006, *I*^2^ = 10%). Subgroup analysis based on the anxiety scale showed the pooled mean difference did not favor either of the 2 groups in the HADS subgroup but favored the PC group in ESAS subgroups (HADS: MD = − 0.24, 95% CI, − 0.96 to 0.48, *p* = 0.52; ESAS: MD = − 0.41, 95% CI − 0.71 to − 0.12, *p* = 0.006) [16]. Li et al. also found that PC interventions significantly reduced anxiety (SMD −0.22, 95% CI −0.40 to −0.05, *I*^2^ = 0%, low quality of evidence) [26]. Kyriakou et al. found that PC had a positive effect on anxiety and trended towards statistical significance (SMD −0.83, 95% CI −3.40 to 1.73, *p* = 0.53) [25].

#### Advanced care planning

ACP was evaluated among four reviews, among these, 3 reviews found that PC intervention improved ACP [17, 27, 28] while one review found no significant difference between groups [21]. Chen et al. found that 3 studies showed improvements in ACP completion after PC intervention. In one study, 94.3% of participants who finished the ACP intervention had a health directive in place, while only 24.8% of those who did not complete the ACP process had a health directive (p < 0.001) [38]. Another study showed a 19% increase in the number of participants completing advanced directives after ACP intervention (*p* = 0.016) [39]. Maqsood et al. found significant increases in ACP following PC intervention (OR 4.29, 95% CI 1.44 to 12.76). Nishikawa et al. found that documentation about the ACP process was completed more often in the intervention group compared with the control group (RR 1.68, 95% CI 1.23 to 2.29; low‐quality evidence). This corresponded to an assumed risk of 489 per 1000 participants with usual care and a corresponding risk of 822 per 1000 participants (95% CI 602 to 1000) for ACP [28]. However, Hicks et al. found that the studies in their review did not demonstrate significantly higher rates of ACP after PC intervention. More advance directives were completed at 3 months in one intervention group, but the significance of differences was not maintained at 6 months [40], nor was there a significant difference between PC and usual care groups in documented ACP or orders for life-sustaining treatment [41].

#### Healthcare utilization

Healthcare utilization was examined in several reviews, to include rehospitalizations and hospital LOS, emergency department (ED) visits, and hospice referrals. 8 out of 10 reviews found that PC intervention reduced rehospitalization [18, 19, 21, 27, 29, 31, 32, 35]. Sebastian et al. found that the PC group experienced a notable decrease in rehospitalizations compared with the usual care group (OR 0.60, 95% CI 0.41 to 0.86, *p* = 0.006, *I*^2^ = 52%) [31]. Maqsood et al. found that PC led to a significant reduction in hospital admissions (OR 0.67, 95% CI 0.48 to 0.95) but was nonsignificant for change in ED visits (OR 0.70, 95% CI 0.38–1.28) [27]. Sahlollbey et al. found that PC interventions were associated with a significant reduction in rehospitalization (OR 0.56, 95% CI 0.33 to 0.94) [29]. Xu et al. also found that PC intervention was associated with a significantly decreased readmission (SMD 0.79, 95% CI 0.23 to 1.35, *p* = 0.006, *I*^2^ = 0%) [32]. In contrast, Zhou et al. found that PC showed no influence on rehospitalization (RR 0.84, 95% CI 0.66 to 1.07, *p* = 0.16), although their pooled analysis only included 2 studies [33]. Hospital LOS was evaluated by one group of investigators. In their review, 5 out of 6 studies showed significant reductions in hospital LOS following PC intervention [18].

Hospice referral was evaluated in two reviews. Datla et al. evaluated 4 studies on hospice use and found that no studies found a significant difference between the PC intervention group and usual care [18]. Likewise, Kim et al. examined 2 studies on hospice use and found that neither study showed significant differences between groups [24].

#### Mortality

Mortality was evaluated within 9 reviews, and 8 reviews described that PC had no significant effect on mortality [16, 18, 21, 26, 29, 31–33] while one review found that those in the ACP group had increased mortality [28]. Almaadawy et al. found that among 3 studies, neither the PC group nor the control group showed a difference regarding mortality under a fixed-effect model (RR 1.11, 95% CI 0.74 to 1.65, *p* = 0.61) [16]. Similarly, Datla et al. evaluated mortality among 8 studies and found that PC interventions did not increase mortality (RR 1.00, 95% CI 0.76 to 1.33, *I*^2^ = 0%, moderate quality of evidence) [18]. Hicks et al. found statistically significantly fewer deaths in the intervention group compared to the control group over a 12-month period in one study (*p* = 0.04) [36], but the remaining 4 studies found no significant difference between groups [21].

Nishikawa et al. evaluated mortality among 5 studies. Pooled analysis showed that mortality may have been increased in participants who received ACP intervention compared with usual care (RR 1.32, 95% CI 1.04 to 1.67, *p* = 0.022, *I*^2^ = 10%). Of the five studies, one study enrolled participants with HF as well as participants with renal disease. A sensitivity analysis including only participants with HF still showed a higher risk of all-cause mortality among the ACP group (RR 1.44, 95% CI 0.99 to 2.09, *p* = 0.058, *I*^2^ = 33%) [28].

#### Satisfaction with care

Satisfaction with care was reported in two reviews. Both reviews found that PC intervention improved patient satisfaction with care. Schichtel et al.’s pooled results indicated that PC intervention was associated with a statistically significant effect (SMD 0.39, 95% CI 0.14 to 0.64, *p* = 0.003, *I*^2 =^ 78%) [30]. Hicks et al. found that 2 out of 3 studies evaluated in their review found statistically significantly higher levels of patient satisfaction with care in the intervention group compared to the control group at 4 weeks and 12 weeks, respectively (*p* = 0.001 and *p* < 0.001) [21].

#### Caregiver burden

Caregiver burden was evaluated in a single review [21]. Caregiver burden was measured by the Zarit Burden Interview instrument (ZBI). A significantly lower caregiver burden was found among the intervention group (post-intervention median caregiver burden score 11.61) compared to the control group at 12 weeks (median caregiver burden score 23, *p* = 0.024) following a PC intervention targeting symptom management, social support, spiritual care, GOC, and end-of-life discussions [42]. Another study in the review demonstrated improved levels of caregiver confidence in providing home care (M − W *z* = 2.8, *p* = 0.003) and reduced caregiver depression (M − W *z* = − 2.4, *p* = 0.01) in the intervention group after 6 months [37].

## Discussion

This is the first umbrella review to comprehensively examine and synthesize 19 systematic reviews incorporating a total of 104 primary studies, on the effect of PC interventions on patient-centered outcomes and resource utilization among patients with HF. Of these systematic reviews, overall methodological quality was high and study overlap was moderate, suggesting a robust and diverse evidence base. Evidence drew from a variety of care settings including the hospital, community, and telehealth, supporting broad applicability of our findings. PC interventions were strongly associated with improvements in relevant patient-centered outcomes, including QOL and depression, ACP, and reduced hospitalizations.

PC was associated with an overall decrease in healthcare utilization, including hospital LOS – this aligns with PC outcomes in other serious illness disease groups including cancer and dementia [43–45]. Interestingly, this umbrella review shows no consistent increase in hospice referrals compared to control groups, a different finding from other studies of chronic illness populations [44, 46, 47]. Prior studies demonstrate that patients with advanced heart disease are referred later to hospice care than other disease groups, often with hospice lengths of stay < 7 days, providing limited time for hospice teams to meaningfully impact the patient and caregiver end-of-life experience [48, 49]. This demonstrates an important opportunity for PC to facilitate hospice referrals in this population.

This umbrella review demonstrates variable findings of the association of PC interventions on the symptomatic experience of patients with HF. The narrative systematic reviews indicated a positive impact on symptom burden due to PC interventions; however, three of the five meta-analyses demonstrated no significant difference between PC and usual care. One reason for these disparate findings may be related to the variety of study measures for symptom assessment that were utilized in the primary studies of the systematic reviews, including: ESAS, GSDS, Memorial Symptom Assessment Scale, Chronic Heart Failure Questionnaire, the HF Symptom Survey, Borg Scale, and multiple instruments for depression and anxiety. Dyspnea and fatigue are common HF-specific symptoms; however, they are variously measured in these instruments and integrated into some of the QOL instruments, which limits the precision with which the impact of PC interventions on symptoms can be evaluated. Furthermore, Li et al. suggest that differences in the PC intervention setting (i.e., hospitalized vs community) may affect findings regarding symptoms due to differences in intervention timing, methods, and care providers. Hospital PC interventions that address symptoms may include more frequent assessments and immediate access to pharmacologic therapies, which may potentially alleviate symptoms more effectively compared to community settings [26].

Most PC interventions had multiple components, integrating domains of care recommended in the AHA/ACC/HFSA HF guidelines, most notably high-quality communication, clarification of GOC, shared decision-making, and symptom management. This is congruent with usual PC practice patterns that concurrently address multidimensional components of serious illness. For patients with advanced HF, care teams often address not only common disease-related physical symptoms (e.g., dyspnea, fatigue, anorexia) [50, 51] but also psychosocial distress [52] and complex communication (e.g., promoting prognostic awareness and facilitating ACP) [53, 54]. This review demonstrates that PC interventions resulted in modest improvements in ACP completion, although when tracked longitudinally, these improvements were not consistently sustained.

There was very little evidence regarding the PC domain of caregiver support. Although attention to caregiver burden abounds in current HF literature and society guidelines [7, 46], only one review evaluated PC’s impact, finding the intervention favorable. Caregivers often share the experience of HF alongside the patient and face unique challenges due to the interdependent care relationship. Caregivers offer emotional support, face prognostic uncertainty, provide care coordination, handle financial issues, and provide physical care to patients for extended periods which can cumulatively contribute to the experience of burden [55]. Future research should characterize opportunities to improve the caregiver experience within PC.

Our umbrella review observed no significant differences in mortality between PC and usual care groups, underscoring that PC for patients with HF enhances quality rather than quantity of life. Other high-quality meta-analyses previously reported no significant effect or uncertain results of PC impacting mortality among patients with life-limiting illness [46, 56]. However, reviews of advanced cancer patients reported that specialty PC was associated with improved survival, and early initiation of PC, received 31 to 365 days after diagnosis, conferred significant survival benefits [57, 58]. Though evidence regarding PC and mortality benefits is mixed and likely context-dependent, PC has not been shown to cause harm or reduce survival, supporting its safety and integration into patient care.

### Practice recommendations

The PC specialty is dedicated to improving QOL for patients, and our findings demonstrate that PC confers consistent benefits for improving depression and ACP as well. Clinical providers should prioritize the implementation of PC to target these key patient-centered outcomes to improve patients’ lived experience with HF, strengthen patient autonomy, and promote value-aligned care. Moreover, our review found strong evidence on the benefits of PC on reducing hospitalizations and LOS, outcomes that are important in clinical practice, and often compelling to policymakers and administrators of healthcare organizations. It is imperative for leaders in PC to establish standards and improve access to high quality PC to maximize the benefits of PC for patients with HF, their families, and the healthcare system.

Primary and specialty PC interventions have often been analyzed together, making it difficult to determine whether specialty PC facilitates greater benefits compared to non-specialty PC interventions. Only one group of investigators in this review performed subgroup analyses based on PC type. Li et al. found that patients receiving specialist PC showed significant improvements in HF-specific QOL, general QOL, and anxiety, while such improvements were not observed in those receiving primary PC. However, between-group differences were not significant for any of the outcomes. Subgroup analyses were inconclusive regarding associations between PC intervention type and outcomes, but findings suggest that specialist PC may offer greater benefits in reducing QOL and anxiety due to specialty PC providers’ advanced specialized training, which may facilitate a more tailored approach to address patients’ complex needs [26].

Clinical practice settings should prioritize more robust and transparent data collection describing the structure, personnel, and processes of PC interventions for patients living with HF and their caregivers. As current integration of PC in HF care is variously implemented in clinical practice and studies vary widely in how interventions are designed and delivered, establishing a standardized taxonomy of PC components – detailing the specific disciplines involved, their relative contributions, and intervention intensity – would improve comparability across studies and care settings. Clear documentation of discipline-specific roles (e.g., nursing, advanced practice providers, medicine, social work, chaplaincy, clinical pharmacy) within multidisciplinary PC teams is especially important to identify which elements and timing derive the greatest benefit for patients and caregivers. Professional guidelines emphasize the need to delineate structure and processes of care and describe team composition and care delivery with precision [59, 60]. Finally, as professional guidelines recommend integration of PC into routine HF care, research and clinical practice guidelines should delineate key points of contact for PC teams to engage with patients along the HF trajectory.

### Policy recommendations

Advancing PC integration into HF management requires healthcare policy initiatives to standardize PC interventions that align with the AHA/ACC/HFSA and National Consensus Project (NCP) for Quality Palliative Care guidelines [59]. The HFSA’s 2025 consensus statement on palliative and supportive care in HF notes the “a lack of consensus…regarding the content, components and standards of PC for patients with HF [7].” Authors emphasize the need for consistent, team-based models of care that delineate processes and points of integration for PC interdisciplinary collaboration, communication, and symptom management. Yet across current research and clinical programs, PC interventions remain heterogeneous in composition, intensity, and delivery. To ensure comparability and scalability, policy and funding mechanisms should support the adoption of standardized intervention frameworks specifying key elements—team composition, frequency and mode of contact, and integration within cardiology and primary care workflows. Embedding these standards into HFSA-endorsed quality metrics and accreditation processes would facilitate consistent implementation across health systems, enable meaningful outcomes benchmarking, and strengthen the evidence base linking PC delivery to improved quality, cost, and patient-centered outcomes in advanced HF. In parallel, harmonizing PC reporting requirements with guideline and NCP recommendations would enhance transparency, reproducibility, and policy relevance, positioning PC as a core component of guideline-directed, high-quality HF management. The success of PC integration into clinical HF care towards achieving outcomes that benefit patient/caregiver and healthcare system ultimately depends on consistent funding mechanisms that support the interdisciplinary team. Currently, while the Centers for Medicare and Medicaid Services (CMS) recognize PC as an important part of HF care – perhaps most notably in the 2020 updated CMS National Coverage Determination for durable left-ventricular assist devices that require a palliative specialist on the care team – no dedicated reimbursement structure for longitudinal interdisciplinary PC exists, outside of the Medicare Hospice Benefit [61]. Provider fee-for-service payment mechanisms are insufficient to bridge this gap, leaving most PC teams without full interdisciplinary representation [62]. Policymakers must develop strategies to fund longitudinal PC that reflect the needs of the HF patient/caregiver illness experience.

## Limitations

There are some important limitations to consider in our review. Primary studies that were evaluated in the systematic reviews had an overall low to very low quality of evidence due to small sample sizes. Larger, longitudinal studies with rigorous study designs are needed. Further, the literature on PC reflects inherently heterogeneous interventions conducted on distinct study populations, limiting generalizability on multiple axes. This reflects the “real-world” experience of clinical PC but correspondingly makes it more difficult to isolate the impact of the PC intervention on clinical outcomes and patient-reported outcomes. The heterogeneity inherent in the HF population, from chronic stable HF to advanced HF, and the various classifications and stages of HF, further limit the ability to generalize conclusions. Nevertheless, our umbrella review aims to synthesize a large and growing body of primary literature through systematic reviews and meta-analyses employing validated measures such as the KCCQ, MLHFQ, and others, to yield some durable conclusions about the impact of PC on patient-reported and healthcare outcomes among patients with HF across a variety of care settings.

### Future studies

Future studies examining the role of PC for patients with HF should examine impact and benefits for specific HF populations known to have a high burden of physical and psychosocial distress and medically complex illness trajectories, including patients living with left ventricular assist devices, heart transplants, cardiac inotropes, pulmonary hypertension, cardiac amyloidosis, adult congenital heart disease, and other such conditions. Pragmatic observational studies, conducted within large health systems to study patients of diverse demographic backgrounds, of early integration of PC for patients with HF with long-term follow-up of patient outcomes, should be explored. PC may have an important role in improving value-based care metrics for patients with serious illnesses. Another area of exploration is the evaluation of the relative impact of primary PC with and without specialty PC on patient and caregiver outcomes. This may include the timing of specialist PC integration, the cadence of longitudinal involvement, the setting of intervention, the modality of intervention, and the key personnel of the interdisciplinary team. Recent methodological papers and reporting frameworks for rigorous capture and standardized reporting of intervention characteristics, intensity, and fidelity have been developed to strengthen reproducibility and synthesis [63–66]. Adhering to these recommendations will allow PC research in HF to evolve from heterogeneous, often single-center findings toward evidence-based, replicable models of integrated, multidisciplinary care. Longitudinal studies are also required, as patients with HF will progress along a HF continuum with associated changes in their PC needs and requirements. Lastly, caregivers were very underrepresented in all of the studies and merit further examination.

## Conclusion

This umbrella review provides a strong foundation of the state of the science of PC interventions and the associated outcomes in the management of patients with HF. Strengths were identified and gaps in the scientific knowledge are described to provide guidance on future directions for practice, policy, and research regarding the provision of PC within HF care. Our findings confirm that PC offers meaningful benefits for patients with HF while identifying critical opportunities to strengthen caregiver inclusion, implementation fidelity, and standardized outcome measurement in future research.

## Supplementary Information

Below is the link to the electronic supplementary material.

Supplementary file1 (DOCX 89 KB)

## Data Availability

No datasets were generated or analysed during the current study.
