# Supplementary material for: Palliative care interventions and outcomes in patients with heart failure: an umbrella review
Source: Heart Fail Rev. 2026 Apr 23;31(1):62. doi: 10.1007/s10741-026-10632-4 (PMC13106263; doi:10.1007/s10741-026-10632-4)
Supplement: Supplementary file 1 — Supplementary file1 (DOCX 89 KB) [file 10741_2026_10632_MOESM1_ESM.docx]

**Table SI. Search Strategy for Appendix**

| **PubMed**  10/17/2025  173 results | (("Systematic Review"[Publication Type] OR ("Systematic”[Text Word] AND “Review"[Text Word]) OR "meta analysis"[Text Word]) AND ("Palliative Medicine"[MeSH Terms] OR "Hospice and Palliative Care Nursing"[MeSH Terms] OR "Palliative Care"[MeSH Terms] OR "Hospice Care"[MeSH Terms] OR "palliati*"[Text Word] OR "PC"[Text Word] OR "end of life"[Text Word] OR "EOL"[Text Word] OR "supportive care"[Text Word] OR "hospice"[Text Word] OR "advance* care planning"[Text Word]) AND ("heart failure"[MeSH Terms] OR "heart failure"[Text Word] OR "HF"[Text Word] OR "CHF"[Text Word] OR "myocardial failure"[Text Word])) |
| --- | --- |
| Filters:  English and Spanish |  |

| **CINAHL**  10/17/2025  123 results | ((MH "Systematic Review") OR XB ( (systematic AND review) OR meta analysis) ) AND  (MH "Heart Failure+") OR XB ( heart failure OR HF OR CHF OR myocardial failure ) AND  ( (MH "Palliative Care") OR (MH "Palliative Care Nursing") OR (MH "Palliative Medicine") ) OR XB ( palliati* OR PC OR end of life OR advance* care planning OR hospice OR supportive care ) |
| --- | --- |
| Filters:  English and Spanish, academic journals |  |

| **Embase**  10/17/2025  334 results | ('systematic review'/exp OR 'review, systematic':ti,ab,kw OR 'systematic review':ti,ab,kw OR ('review':ti,ab,kw AND 'systematic':ti,ab,kw) OR 'meta analysis'/exp OR 'analysis, meta':ti,ab,kw OR 'meta analysis':ti,ab,kw OR 'meta-analysis':ti,ab,kw OR 'metaanalysis':ti,ab,kw) AND ('heart failure'/exp OR 'backward failure, heart':ti,ab,kw OR 'cardiac backward failure':ti,ab,kw OR 'cardiac decompensation':ti,ab,kw OR 'cardiac failure':ti,ab,kw OR 'cardiac incompetence':ti,ab,kw OR 'cardiac insufficiency':ti,ab,kw OR 'cardiac stand still':ti,ab,kw OR 'cardial decompensation':ti,ab,kw OR 'cardial insufficiency':ti,ab,kw OR 'chronic heart failure':ti,ab,kw OR 'chronic heart insufficiency':ti,ab,kw OR 'decompensatio cordis':ti,ab,kw OR 'decompensation, heart':ti,ab,kw OR 'heart backward failure':ti,ab,kw OR 'heart decompensation':ti,ab,kw OR 'heart failure':ti,ab,kw OR 'heart incompetence':ti,ab,kw OR 'heart insufficiency':ti,ab,kw OR 'insufficientia cardis':ti,ab,kw OR 'myocardial failure':ti,ab,kw OR 'myocardial insufficiency':ti,ab,kw OR 'hf':ti,ab OR 'chf':ti,ab) AND ('palliative therapy'/exp OR 'palliation':ti,ab,kw OR 'palliative care':ti,ab,kw OR 'palliative consultation':ti,ab,kw OR 'palliative medicine':ti,ab,kw OR 'palliative radiotherapy':ti,ab,kw OR 'palliative surgery':ti,ab,kw OR 'palliative therapy':ti,ab,kw OR 'palliative treatment':ti,ab,kw OR 'symptomatic treatment':ti,ab,kw OR 'palliative nursing'/exp OR 'hospice and palliative care nursing':ti,ab,kw OR 'palliative care nursing':ti,ab,kw OR 'palliative nursing':ti,ab,kw OR 'palliati*':ti,ab OR 'hospice'/exp OR 'hospice':ti,ab,kw OR 'advance* care planning':ti,ab,kw OR 'hospices':ti,ab,kw OR 'terminal care'/exp OR 'eol care':ti,ab,kw OR 'end-of-life care':ti,ab,kw OR 'terminal care':ti,ab,kw) |
| --- | --- |
| Filters:  English and Spanish, review, article, article in press |  |

**Table SII. Table of Included Study Characteristics**

| **Authors, year,**  **type of systematic review, country** | **Objective** | **Number, type and publication date of studies** | **Countries and settings** | **Sources and language restrictions** | **Participants (diagnosis, age, number** | **Quality assessment instrument and rating of studies** | **Intervention and type of PC** | **Outcomes and Measures** | **Results** |
| --- | --- | --- | --- | --- | --- | --- | --- | --- | --- |
| Agho et al., 2025 [15]  Systematic review and narrative synthesis  USA | To evaluate how integrating palliative care into heart failure management enhances patient quality of life (QOL) by addressing symptom control, improving care satisfaction, and ensuring informed decision-making throughout the disease trajectory. | **Number of Studies**  18 studies  **Type**  RCT=10  Cohort=3  Observational=1  Clinical practice guideline=1  Implementation study=1  Qualitative=1  **Publication Dates**  2007-2024 | Countries and settings not reported | Embase, PubMed, SCOPUS, Web of Science, and Google Scholar from 2000-2025  English language only | Adults with HF  7604 total participants | **Quality assessment instrument**  AXIS  **Quality rating**  Low rating=0 studies  Moderate rating=6 studies  High rating=12 studies | Focus on PC (primary and specialty PC) in patients with HF | QOL  Measures not reported | PC is effective in improving QOL among patients with HF |
| Almaadawy et al., 2025 [16]  Systematic review and meta-analysis  USA | To systematically evaluate the impact of palliative care interventions on QOL, symptom burden, and psychological well-being in patients with advanced HF. | **Number of Studies**  13 studies  **Type**  RCT=13  **Publication Dates**  2011-2023 | **Countries**  USA (n=6)  China (n = 4) Iran (n = 2)  Sweden (n =1)  **Settings**  Hospital, home, telehealth, telephone | PubMed, Cochrane, Web of Science, Embase, and Scopus  English language only | Participants diagnosed with HF  18 years or older  1541 total participants | **Quality assessment instrument**  RoB 2  **Quality rating**  Low risk of bias= 4 studies  Some concerns=8 studies  High risk of bias=1 study | PC intervention compared with a usual care group  Does not indicate type of PC | -QOL (KCCQ, ESAS, ED5Q, FACIT-Pal, PHQ-9, MLHFQ)  -Mortality  -Symptoms (GSDS, Borg Scale)  -Anxiety (GAD-7)  -Depression (PHQ-9, PHQ-4, HADS) | PC significantly improved QOL compared with usual care (MD = 3.59; 95% CI, 0.64–6.45; *P* = 0.02; *I*^2^ = 95%). After removing an outlier study, the mean difference still favored the PC group (MD 1.36, 95% CI 0.89 to 1.83, p < 0.00001, I^2^ = 0%).  PC significantly reduced anxiety scores (MD -0.39, 95% CI -0.66 to -0.11, p = 0.006, I^2^ = 10%).  No significant differences were observed for dyspnea (MD -0.15, CI -0.66 to 0.36, p=0.56, I^2^ = 78%), or depression (MD = −0.34; 95% CI, −0.91 to 0.22; *P* = 0.23; *I*^2^ = 0%).  Mortality rates did not differ significantly between groups (RR 1.11, 95% CI 0.74 to 1.65, p = 0.61). |
| Chen et al., 2025 [17]  Mixed-methods systematic review and meta-analysis  China | To integrate the data on the effects of a pre-established medical care program on hospitalized older adults with chronic HF. | **Number of Studies**  11 studies  **Type**  RCT=5  Quasi-experimental=3  Mixed method=1  Qualitative=2  **Publication Dates**  2011-2024 | **Countries**  Countries not reported  **Settings**  Hospital | PubMed, CINAHL, MEDLINE, Web of Science, CNKI, Embase, and Cochrane Library (through October 29, 2024) | Patients diagnosed with HF  Ages 50 or older  2809 total participants | **Quality assessment instrument**  JBI Critical Appraisal Tool  **Quality rating**  Low quality=2 studies  Medium quality=6 studies  High quality=1 study | Interventions focused on ACP or advance directives, with or without a comparison group | -ACP completion  -Satisfaction with care  Measures not reported | Results could not be statistically summarized and were narratively reviewed.  Three studies showed ACP-related completion after ACP intervention  ACP can significantly increase the proportion of patients to receive hospice services |
| Datla et al., 2019 [18]  Systematic review and narrative synthesis  UK | To identify the evidence in relation to palliative care for people with symptomatic HF. | **Number of studies**  23 studies  **Type**  RCT=8  Pilot study=4  Quasi-experimental=1  Cohort study=7  Case-control =2  Cross-sectional=1  **Publication dates**  2005-2017 | **Countries**  United States (n=16),  United Kingdom (n=3), Sweden (n=2), China (n=1), Singapore (n=1)  **Settings**  Community, hospital, hospice | Medline, Cochrane database, CINAHL, PsycINFO, HMIC, and Care Search Grey Literature (1995- March 31, 2019)  No language restrictions | Participants with HF NYHA III-IV or advanced HF  19891 total participants | **Quality assessment instrument**  RoB  Newcastle-Ottawa quality assessment scale    **Quality rating**  All studies had high risk of bias for blinding and outcomes | Any study with a component of PC intervention delivered by a specialized or generalist service apart from ACP alone | -Symptoms (ESAS, MSAS)  -Depression (PHQ-9, HADS, QIDS)  -QOL (KCCQ, EQ5D)  -Mortality  -Hospitalization | Multidisciplinary specialist PC intervention showed statistically significant benefits for symptom burden, depression, QOL  4 out of 5 studies found no significant difference in mortality between the intervention and control group  PC interventions showed statistically significant reductions in rehospitalizations and hospital length of stay  There was no significant difference in hospice use among participants |
| DeGroot et al., 2020 [19]  Systematic Review and narrative synthesis  USA | To synthesize the literature of outpatient PC in HF to identify the current landscape, the impact on patient health outcomes, key stakeholders' perspectives, and future implications for research and practice. | **Number of studies**  19 studies  **Type**  RCT=6  Prospective=1  Descriptive exploratory=1  Descriptive correlational=1  Chart review=3  Qualitative=6  Mixed methods=1  **Publication dates**  2006-2018 | **Countries**  USA (n=10), Sweden (n=6),  China (n=3)  **Settings**  Home, community, hospital, telephone, online survey | PubMed, Embase, CINAHL, Cochrane, and Web of Science (inception to February 2019)  English language only | Participants with HF, informal caregivers of adult HF patients, or HF providers  Ages 18 years or older  1231 total participants | **Quality assessment instrument**  Johns Hopkins Nursing Evidence-Based Practice Appraisal tool  **Quality rating**  High quality (A)=6  Good quality (B) =12  Low quality (C)=1 | Primary or specialty PC provided as an intervention or service  Most studies utilized specialty PC interventions | -Symptoms (ESAS)  -QOL (KCCQ, MQOL)  -Depression  -Anxiety  -ACP | Outpatient PC improved QOL, alleviated symptoms, and decreased rehospitalization |
| Fernandes Pedro & Reis-Pina, 2022 [20]  Systematic review and narrative synthesis  Portugal | To systematically review the efficacy of integrating PC in patients with advanced HF, including the outcomes overall quality of life and well-being, overall symptom burden and possible specific symptoms, hospital admission rates and mortality. | **Total number of studies**  7 studies  **Type**  RCT=4  Cohort=1  Prospective=2  **Publication dates**  2014-2018 | Countries and settings not specified | MEDLINE, Cochrane, EMBASE and CINAHL databases were searched for articles published between January 2010 and December 2020  Language restrictions not specified | Participants with HF (NYHA class II or higher)  No age restrictions  5388 total participants | **Quality assessment instrument**  Cochrane tools for RCT  ROBINS-I  **Quality rating**  Overall risk of bias within studies was moderate to high | PC interventions (primary or specialty PC) with assessment of symptom burden, QOL, hospital readmission, or mortality, compared to usual care | -QOL  -Symptoms  -Hospital readmission  Measures not reported | Most studies showed significant improvements in QOL and symptoms  Mixed evidence showing improvements in hospital readmission between groups |
| Hicks et al., 2022 [21]  Systematic Review and narrative synthesis  UK | To provide an evidence synthesis of the effectiveness and cost-effectiveness of PC interventions for people with chronic HF and their caregivers.” | **Number of Studies**  18 studies  **Type**  RCT=17  Mixed methods=1  **Publication dates**  1998-2019 | **Countries**  USA (n=9)  Sweden (n=5),  China (n=3),  UK (n=1)  **Settings**  Community, outpatient, hospital, mixed | MEDLINE, Embase, PsychInfo, CINAHL, CENTRAL, and HMIC (inception to June 2019) | Participants with a diagnosis of CHF or NYHA II-IV as a primary or secondary diagnosis  Ages 18 years or older  1817 total participants | **Quality assessment instrument**  RoB 2  **Quality rating**  Low risk of bias (n=4)  Some concerns (n=4)  High risk of bias (n=10) | Two or more clinical or non-clinical PC interventions delivered by primary or specialty PC teams | -QOL (CHQ-C, EQ5D, FACIT-PAL, KCCQ, MLHFQ)  -Symptoms (GSDS)  -Psychological status (BDI, GAD-7, HADS, PHQ-9)  -Satisfaction with care  -Mortality  -ACP  -Rehospitalization  -Caregiver burden (ZBI) | PC interventions showed greatest evidence for benefit for QOL, psychological status, satisfaction with care, symptom burden, reductions in hospitalization, and caregiver burden  4 out of 5 studies showed no significant difference in mortality between groups  No significant differences found for ACP between groups |
| Kane et al., 2015 [22]  Systematic review and narrative synthesis  UK | To identify patient-centered care (PCC) interventions in CHF where patients’ are involved as informed, active participants in shared decision making about their clinical care and identify their own personal care goals and to describe domains of PCC included in the interventions and to describe the selected outcomes. | **Total number of studies**  10 studies  **Type**  RCT=3  Feasibility=2  Before and after=2  Prospective=2  Retrospective=1  **Publication dates**  2005-2014 | **Countries**  USA (n=8)  Sweden (n=2)  **Settings**  Hospital, community | Medline, Embase, PsycINFO, CINAHL, ProQuest ASSIA, Cochrane databases and clinicaltrials.gov were searched from inception to March 2015  English language only | Participants with chronic HF, NYHA class II-IV  18 years or older  2540 total participants | **Quality assessment instrument**  Down and Black checklist for RCTs and non-RCTs  **Quality rating**  Median quality score was 20 (possible total score of 32) | Intervention aimed to increase patient-centered care behavior by incorporating shared decision-making, involving one or more of: promoting patient participation in care plan formulation, shared control of the patient-provider consultation, patient self-identification of their goals of care, delivered by primary or specialty PC providers | -QOL (KCCQ, MLHFQ)  -Symptoms (ESAS)  -Depression (PHQ-9) | Though the strength of evidence is poor, PCC reduced symptom burden, depression, and improved QOL |
| Kernick et al., 2018 [23]  Systematic review and narrative synthesis  UK | To assess whether ACP, in addition to usual care, reduces the number of hospital admissions for patients with advanced HF. To assess whether ACP, in addition to usual care, improves adherence to patient preferences in care, patient-reported outcomes, place of death and satisfaction with care. | **Total number of studies**  8 studies  **Type**  RCT=4  Observational=4  **Publication dates**  2011-2017 | **Countries**  USA (n=3)  UK (n=2)  Canada (n=1)  Sweden (n=1)  Hong Kong (n=1)  **Settings**  Community, hospital | MEDLINE, Cochrane Library, CINAHL and Scopus from 1990 to March 23 2017 | Participants with all causes and classifications of HF  14357 total participants | **Quality assessment instrument**  RoB or the CASP Tool for Cohort Studies  **Quality rating**  Moderate quality among RCTs | Interventions containing ACP/directive, living will, medical directive, resuscitation plan, end of life plan, anticipatory care plan or medical treatment plan, compared with usual care, delivered by primary or specialty PC providers | -Hospital admissions  -QOL (MQOL, KCCQ, FACIT-Pal)  -Symptoms (ESAS, CHQ-Chinese, MQOL-Chinese) | ACP interventions led to statistically significant reductions in hospital readmission and increased use of palliative services |
| Kim et al., 2022 [24]  Integrative review  South Korea | To contribute to the basic design of future intervention programs by identifying and reviewing the specific aspects of PC interventions reported in trials for patients with HF. | **Total number of studies**  6 studies  **Type**  RCT=6  **Publication dates**  2014-2016 | **Countries**  USA (n=4)  Sweden (n=1)  Hong Kong (n=1)  **Settings**  Hospital, home, telephone | RISS, KoreaMed, Korean Medical Database (KMbase), CINAHL, PubMed, MEDLINE, and Cochrane Library CENTRAL  Studies published in English and Korean | Adults with HF  Total number of participants not reported | **Quality assessment instrument**  RoB  **Quality rating**  Overall quality assessment not provided | Multidisciplinary team-based primary or specialty PC interventions compared with usual care | -QOL (KCCQ, MLHFQ, EQ5D, MQOL, CHFQ, FACIT-Pal)  -Symptom burden (ESAS)  -Depression (HADS)  -Hospital readmission  -ACP documentation  -Mortality  -Hospice referral | Most studies showed statistically significant improvements in QOL, depression and symptom burden following PC intervention  Studies did not show significant differences between groups in hospice use |
| Kyriakou et al., 2020 [25]  Systematic review and meta-analysis  Cyprus | To examine the effectiveness of supportive care interventions in improving the HRQoL of patients with HF versus a control group. | **Total number of studies**  10 studies  **Type**  RCT=10  **Publication dates**  2004-2015 | **Countries**  USA (n=1)  UK (n=2)  Taiwan (n=1)  Greece (n=2)  Iran (n=1)  Russia (n=1)  Spain (n=1)  Netherlands (n=1)  **Settings**  Community, home | PubMed, CINAHL, and the Cochrane Library up to March 2017  English language only | Adults with HF  3144 total participants | **Quality assessment instrument**  CONSORT 2010 checklist  **Quality rating**  9 trials reported more than half of the checklist items | Supportive care interventions (including any components of communication, education, psychosocial/spiritual, or symptom management) compared to usual care | -QOL (MLHFQ, SF-36, KCCQ, Quality of Life Index)  -Depression (HADS, BDI)  -Anxiety (HADS) | Overall effect indicated a positive effect of supportive care on QOL (SMD -9.44, 95% CI 15.54 to -3.33, p = 0.002).  Supportive care was found to have a positive effect on depression (SMD −0.53; 95% CI, −1.23 to 0.16, p=0.13) and anxiety (SMD -0.83, 95% CI -3.40 to 1.73, p=0.53) but was not statistically significant for either outcome |
| Li et al., 2024 [26]  Systematic review and meta-analysis  China | To evaluate the effectiveness of PC interventions on patient-reported outcomes and all-cause mortality in community-dwelling adults with HF. | **Total number of studies**  11 studies  **Type**  RCT=11  **Publication dates**  2006-2023 | **Countries**  USA (n=7)  Sweden (n=1)  UK (n=1)  Iran (n=1)  Hong Kong (n=1)  **Settings**  Hybrid (face-to-face and telehealth), home, hospital | MEDLINE, Embase, Cochrane Library, and CINAHL from inception to October 2023  English language only | Community-dwelling adults with a primary diagnosis of HF  18 years or older  1535 total participants | **Quality assessment instrument**  RoB 2 and GRADE approach  **Quality rating**  8 studies evaluating QOL rated as having a high risk of bias  Overall low to moderate quality | Primary or specialty PC intervention compared with usual care, attention control, or waiting-list group | -QOL (SF-36, FACIT-Pal, KCCQ, EQ5D, QOL Index, MLHFQ, MQOL, CHFQ)  -Symptoms (ESAS, HF Symptom Survey)  -Anxiety (GAD, HADS, Kessler Scale)  -Depression (HADS, PHQ-9)  -Mortality | After excluding an outlier study, PC interventions demonstrated statistically significant effects on improving health-related QOL (SMD 0.30, 95% CI 0.12 – 0.48; *I*^2^ = 0 %; moderate quality of evidence) and HF-specific QOL (SMD 0.17, 95% CI 0.03 – 0.31; *I*^2^ = 0 %; low quality of evidence).  PC interventions reduced anxiety (SMD -0.22, 95% CI -0.40 to -0.05; *I*^2^ = 0 %; low quality of evidence) and depression (SMD -0.18, 95% CI -0.33 to -0.03; *I*^2^ = 20 %; low quality of evidence).  PC interventions did not adversely affect mortality (RR 1.00, 95% CI 0.76 to 1.33)  PC interventions had no significant effects on symptom burden (SMD, − 0.09; 95 % CI, − 0.40 to 0.21; *I*^2^ = 0 %; low quality of evidence). |
| Maqsood et al., 2021 [27]  Systematic review and meta-analysis  USA | To assess a difference in outcomes in trials with HF patients exclusively vs studies of other noncancer chronic illness following PC intervention | **Total number of studies**  10 studies on patients with HF  **Type**  RCT=10  **Publication dates**  2002-2019 | **Countries**  USA (n=6)  Canada (n=2)  Sweden (n=1)  Hong Kong (n=1)  **Settings**  Hospital and emergency department | PubMed, EMBASE, and grey literature search until August 9 2020  Language restrictions not specified | Patients with HF  18 years or older  4057 total participants | **Quality assessment instrument**  GRADE system  **Quality rating**  High certainty of evidence across studies | PC intervention (type of PC not specified) compared to usual care | -QOL  -Hospitalization  -ACP  -Symptoms  Measures not reported | PC led to a significant reduction in hospital admissions (OR 0.67, 95% CI 0.48 to 0.95; *I*^2^=45.96% )  No significant increase in QOL was seen after PC intervention (WMD 0.04, 95% CI -1.37 to 1.45)  PC intervention was nonsignificant for change in ED visits (OR 0.70, 95% CI 0.38 to 1.28)  PC intervention led to increases in ACP (OR 4.29, 95% CI 1.44 to 12.76)  There was a nonsignificant  improvement in symptom burden following PC intervention (WMD=-3.05; 95% CI = -7.15 to 1.04) |
| Nishikawa et al., 2020 [28]  Systematic review and meta-analysis  Japan | To assess the effects of advance care planning (ACP) in people with HF compared to usual care strategies that do not have any components promoting ACP. | **Total number of studies**  9 studies  **Type**  RCT=9  **Publication dates**  2004-2018 | **Countries**  USA (n=8)  UK (n=1)  **Settings**  Hospital, community | CENTRAL, MEDLINE, Embase, CINAHL, Social Work Abstracts, clinical trial registers until October 2019  No language restrictions | Adults with any type of HF  18 years or older  1242 total participants | **Quality assessment instrument**  GRADE approach  **Quality rating**  Very low/low quality | ACP interventions (considering participant values, wishes, goals; understanding participants’ prognosis; preferences for care) compared with usual care | -QOL (KCCQ, MLHFQ, EQ5D, FACIT-Pal)  -Depression (PHQ-8, PHQ-9, HADS)  -Hospice use  -ACP  -Mortality | No difference was observed in QOL between ACP and usual care (SMD 0.06, 95% CI –0.26 to 0.38; p = 0.71; low-quality evidence)  Documentation about ACP was more often completed in the ACP group compared with usual care group (RR 1.68, 95% CI 1.23 to 2.29; p = 0.0011; low quality evidence)  ACP may have improved depression compared with usual care (SMD –0.58, 95% CI –0.82 to –0.34, low‐quality evidence  All-cause mortality might have increased in participants who received ACP intervention compared with usual care (RR 1.44, 95% CI 0.99 to 2.09, p = 0.058) |
| Sahlollbey et al., 2020 [29]  Systematic review and meta-analysis  Canada | To examine the impact of PC on acute care hospitalization, survival, symptoms, and QOL in patients with advanced HF. | **Total number of studies**  10 studies  **Type**  RCT=10  **Publication dates**  2006-2019 | **Countries**  USA (n=10)  **Settings**  Hospital, outpatient, mixed | CENTRAL, CINAHL, EMBASE, MEDLINE up to June 2019  English language only | Adults with advanced HF (studies of mixed chronic disease)  18 years or older  1050 total participants (87.7% with HF) | **Quality assessment instrument**  Cochrane RoB Tool  **Quality rating**  All studies had a high risk for performance-related bias and detection bias for subjective outcomes | Interdisciplinary PC interventions (primary and specialty PC) compared to usual care in a HF population | -QOL  -Symptom burden  -Hospitalizations  -Mortality  Measures not reported | Compared with usual care, PC interventions were associated with a significant reduction in hospitalization (OR 0.56, 95% CI 0.33 to 0.94; I*2* = 27%), modest improvements in QOL (SMD 0.25, 95% CI 0.06 to 0.45; *I2=* 15%), and modest reduction in symptom burden (SMD -0.29, 95% CI -0.54 to 0.03; *I2* = 15%).  There was no clear adverse impact on mortality with PC interventions (OR 1.30, 95% CI 0.80–2.09; *I2* = 33%). |
| Schichtel et al., 2020 [30]  Systematic review and meta-analysis  UK | To determine whether end-of-life care which included ACP resulted in improved outcomes for patients suffering from HF compared to end-of-life care which did not incorporate ACP. | **Total number of studies**  14 studies  **Type**  RCT=14  **Publication dates**  2004-2017 | **Countries**  USA (n=10)  Sweden (n=1)  UK (n=1)  Australia (n=1)  Hong Kong (n=1)  **Settings**  Community, hospital | CINAHL, Cochrane Central Register of Controlled Trials, Cochrane Database of Systematic Reviews, Database of Abstracts of Reviews of Effects, Embase, ERIC, MEDLINE, Other Non-Indexed Citations and Ovid MEDLINE(R), Science Citation Index, Social Science Citation Index & Conference Proceedings, PsycINFO  Language restrictions not specified | Adults with HF treated in a hospital, hospice or community setting  2924 total participants | **Quality assessment instrument**  Cochrane RoB tool and GRADE tool  **Quality rating**  Mean score across all studies was low to moderate | All types of ACP interventions (primary and specialty PC) that provided a coordinated and comprehensive approach of care for patients early, during or towards the end of suffering from HF | -QOL  -Satisfaction with care  Measures not reported | ACP was associated with a statistically significant improvement for QOL (SMD, 0.38; 95% CI 0.09 to 0.66, *p* < 0.009; *I^2^=*72%).  ACP was associated with a statistically significant effect on patient satisfaction (SMD 0.39, 95% CI 0.14 to 0.64, *p* = 0.003; *I^2^*=78%). . |
| Sebastian et al., 2024 [31]  Systematic review and meta-analysis  India | To analyze the impact of PC telehealth intervention compared to usual care in QOL and resource utilization of chronic HF patients. | **Total number of studies**  16 studies  **Type**  RCT=16  **Publication dates**  2014-2024 | **Countries**  USA (n=7)  UK (n=1)  India (n=1)  China (n=2)  Iran (n=2)  Poland (n=1)  Turkey (n=1)  Sweden (n=1)  **Settings**  Home, hospital | MEDLINE (via PubMed), Google Scholar, the Cochrane Library, and ScienceDirect  Until May 2024  Language restrictions not specified | Adults with chronic HF  Ages 18 years or older  2324 total participants | **Quality assessment instrument**  RoB 2  **Quality rating**  Overall bias assessment suggested low risk of bias | Primary or specialty PC or palliative telehealth interventions compared to usual care | -QOL (KCCQ, FACIT-Pal)  -Hospitalization  -Mortality | Improvements in QOL among patients who received PC or palliative care telehealth interventions compared to those who received usual care. WMD for KCCQ was 3.56 (95% CI 0.43 to 6.69, p = 0.03; *I* ^2^ = 46%) and for FACIT-Pal was 2.54 (95% CI 1.00 to 4.08, p = 0.001; *I*^2^ = 14%).  Palliative care experienced a notable decrease in hospitalizations (OR 0.60, 95% CI 0.41 to 0.86, p = 0.006, *I*^2^ = 52%)  There was no significant change in all-cause mortality OR 1.22, 95% CI 0.77 to 1.94, p = 0.39, *I*^2^ = 37%) |
| Xu et al., 2018 [32]  Systematic review and meta-analysis  China | To evaluate the effectiveness of PC in patients undergoing HF. | **Total number of studies**  5 studies  **Type**  RCT=5  **Publication dates**  2012-2016 | Countries and settings not reported | PubMed, Embase, Web of Science, EBSCO, and the Cochrane library were systematically searched from inception to May 2017  Language restrictions not specified | Patients diagnosed with HF  Ages not specified  545 total participants | **Quality assessment instrument**  Jadad Scale (a score >3 is considered to be high quality)  **Quality rating**  Scores ranged from 3 to 5 | PC interventions (primary or specialty PC not specified) versus usual care | -Hospital readmission  -QOL (MLHFQ)  -Symptoms (ESAS)  -Depression (PHQ-9)  -Mortality | PC intervention was associated with a significantly decreased readmission (SMD 0.79, 95% CI 0.23 to 1.35, P=0.006; I^2^=0%).  PC was not associated with decreased mortality (RR 1.54, 95% CI 0.80 to 2.96, P = 0.19; I^2^=0%).  PC intervention showed significantly reduced symptoms (SMD -2.5, 95% CI -4.39 to -0.62, P= 0.009) and depression (SMD -1.16, 95% CI -1.73 to -0.58, P< 0.005  PC had no influence on quality of life (SMD 1.81, 95% CI -0.14 to 3.7, P=0.07) |
| Zhou & Mao, 2019 [33]  Systematic review and meta-analysis  China | To compare the efficacy and safety of PC in patients with HF | **Total number of studies**  7 studies  **Type**  RCT=7  **Publication dates**  2008-2017 | Countries and settings not reported | PubMed, Embase, Web of Science, EBSCO, and the Cochrane Library until August 2017 | Adults with HF  769 total participants | **Quality assessment instrument**  Jadad scale  **Quality rating**  Scores ranged from 3-5 | PC intervention (primary or specialty PC not specified) versus usual care | -QOL  -Depression  -Mortality  -Rehospitalization  Measures not reported | PC was associated with a significantly increased quality of life (SMD = 1.46; 95% CI = 0.12 to 2.79; p = 0.03; I^2^=96%) and reduced depression scores (SMD = -0.62; 95% CI = -0.99 to -0.25; p = 0.03; I^2^=24%)  PC demonstrated no impact on mortality (RR 1.28, 95% CI 0.86 to 1.92, p = 0.22; *I*^2^ = 0%).  PC showed no influence on  rehospitalization (RR 0.84, 95% CI 0.66 to 1.07, p = 0.16; I^2^=0%). |

AD, advanced directive; ACP, advanced care planning; AXIS, Appraisal tool for cross-sectional studies; BDI, Beck’s Depression Inventory; CASP, Critical Appraisal Skills Programme; CHFQ, Chronic Heart Failure Questionnaire; CHQ-C, Chronic Heart Failure Questionnaire- Chinese; CONSORT, Consolidated Standards Of Reporting Trials; ESAS, Edmonton Symptom Assessment Scale; EQ5D, EuroQol-5D; FACIT-PaL, Functional Assessment of Chronic Illness Therapy- Palliative Care scale; GAD-7, Generalized Anxiety Disorder Questionnaire; GRADE, Grading of Recommendations Assessment, Development, and Evaluation; GSDS, General Symptom Distress Scale; HADS, Hospital Anxiety and Depression Survey; HF, heart failure; HRQOL-14, Health-Related Quality of Life Scale; JBI, Joanna Briggs Institute; KCCQ, Kansas City Cardiomyopathy Questionnaire; NYHA, MLHFQ, Minnesota Living with Heart Failure Questionnaire; MSAS, Memorial Symptom Assessment Scale; MQOL, McGill Quality of Life Questionnaire; New York Heart Association; PC, palliative care; PHQ-4/PHQ-9, Patient Health Questionnaire, QIDS, Quick Inventory of Depressive Symptomology; QOL, quality of life; RCT, randomized controlled trial; RoB, Revised Cochrane Risk of Bias Tool; ROBINS-I, Risk Of Bias In Non-randomized Studies - of Interventions; SF-36, 36-Item Short Form Survey; ZBI, Zarit Burden Interview Instrument

**Table SIII. ROBIS Assessment**

|  | **Phase 2** | | | | **Phase 3** |
| --- | --- | --- | --- | --- | --- |
| **Review** | **1. Study eligibility criteria** | **2. Identification and selection of studies** | **3. Data collection and study appraisal** | **4. Synthesis and findings** | **Risk of bias in the review** |
| Agho (2025) [15] | 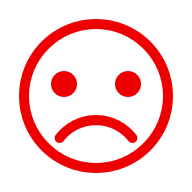 | 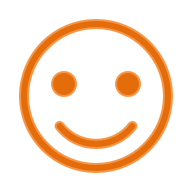 | 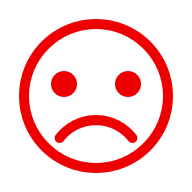 | 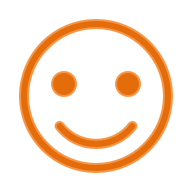 | 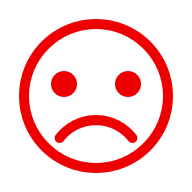 |
| Almaadawy (2025) [16] | 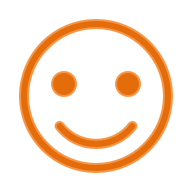 | 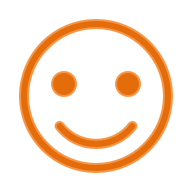 | 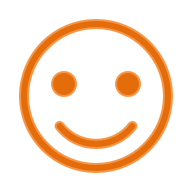 | 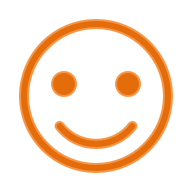 | 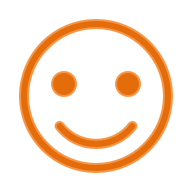 |
| Chen (2025) [17] | 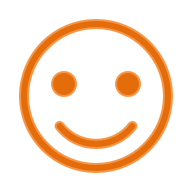 | 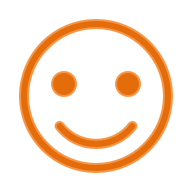 | 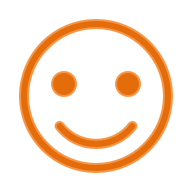 | 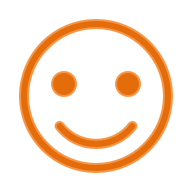 | 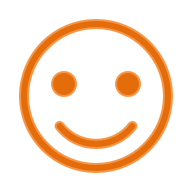 |
| Datla (2019) [18] | 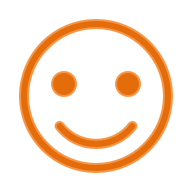 | 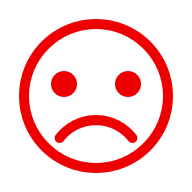 | 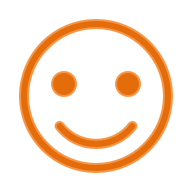 | 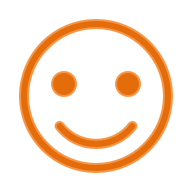 | 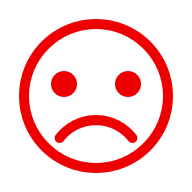 |
| DeGroot (2020) [19] | 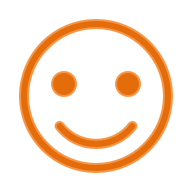 | 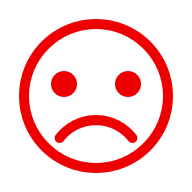 | 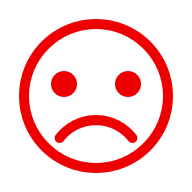 | 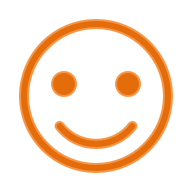 | 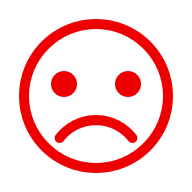 |
| Fernandes Pedro (2022) [20] | 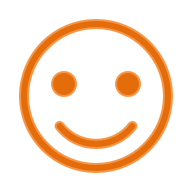 | 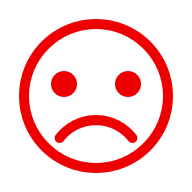 | 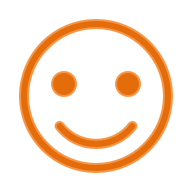 | 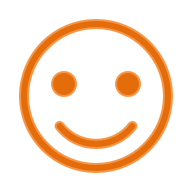 | 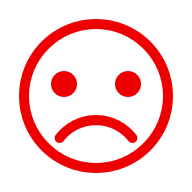 |
| Hicks (2022) [21] | 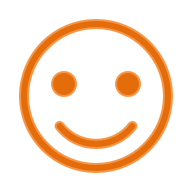 | 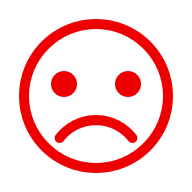 | 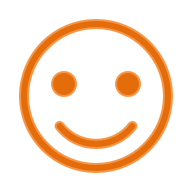 | 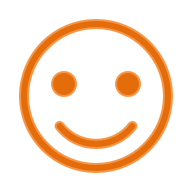 | 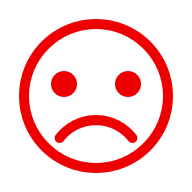 |
| Kane (2015) [22] | 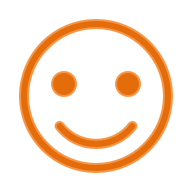 | 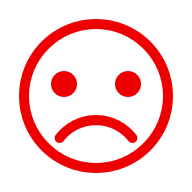 | 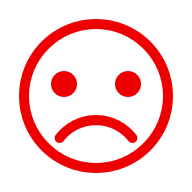 | 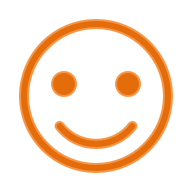 | 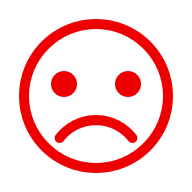 |
| Kernick (2018) [23] | 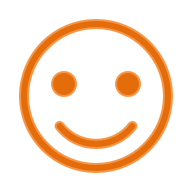 | 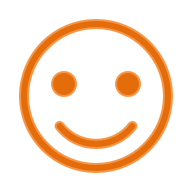 | 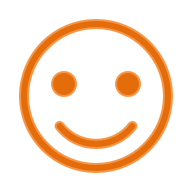 | 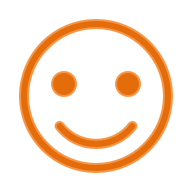 | 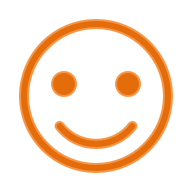 |
| Kim (2022) [24] | 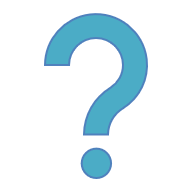 | 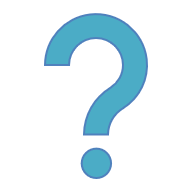 | 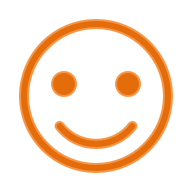 | 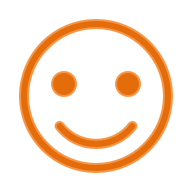 | 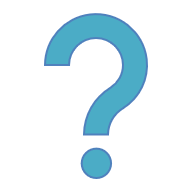 |
| Kyriakou (2021) [25] | 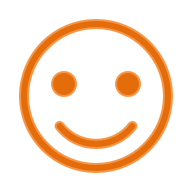 | 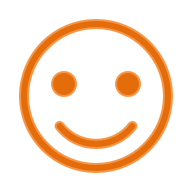 | 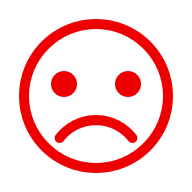 | 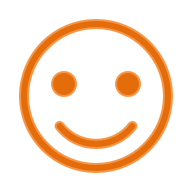 | 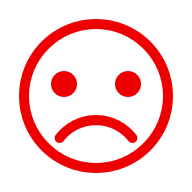 |
| Li (2024) [26] | 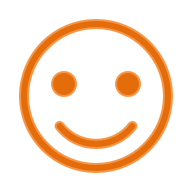 | 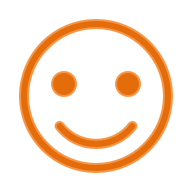 | 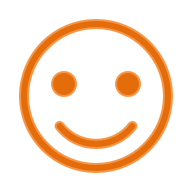 | 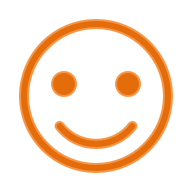 | 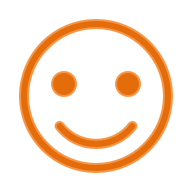 |
| Maqsood (2021) [27] | 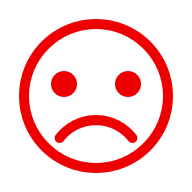 | 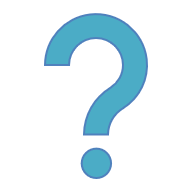 | 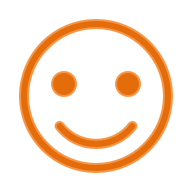 | 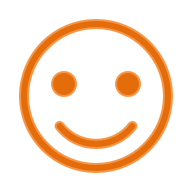 | 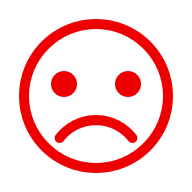 |
| Nishikawa (2020) [28] | 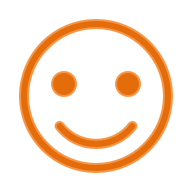 | 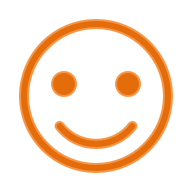 | 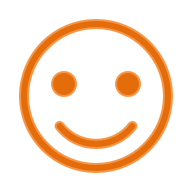 | 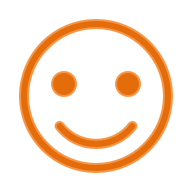 | 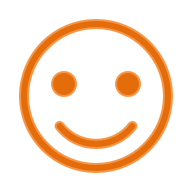 |
| Sahlollbey (2020) [29] | 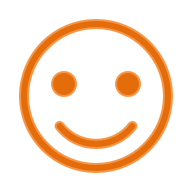 | 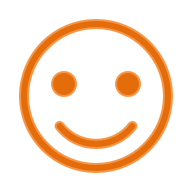 | 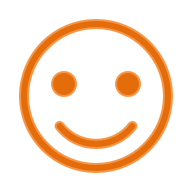 | 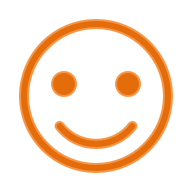 | 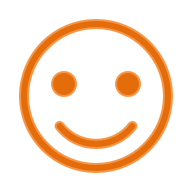 |
| Schichtel (2020) [30] | 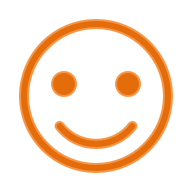 | 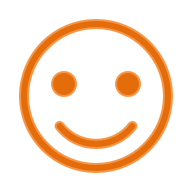 | 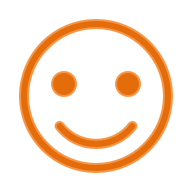 | 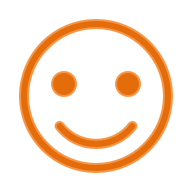 | 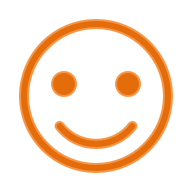 |
| Sebastian (2024) [31] | 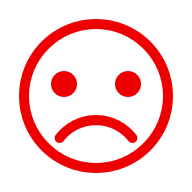 | 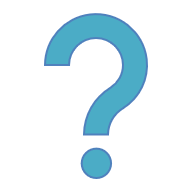 | 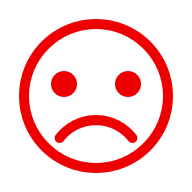 | 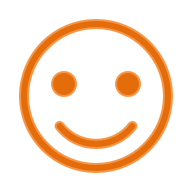 | 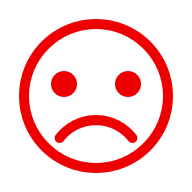 |
| Xu (2018) [32] | 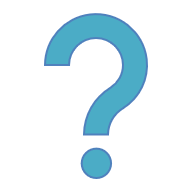 | 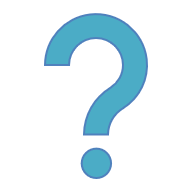 | 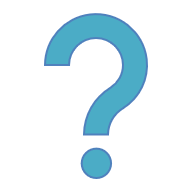 | 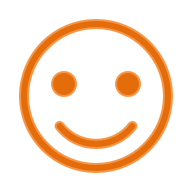 | 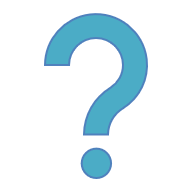 |
| Zhou (2019) [33] | 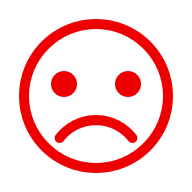 | 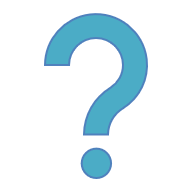 | 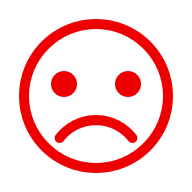 | 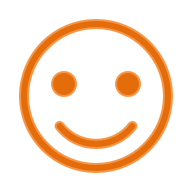 | 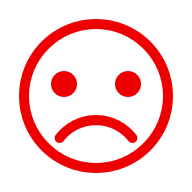 |


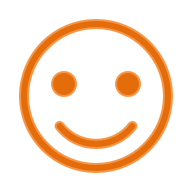
= low risk;
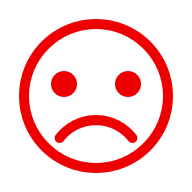
= high risk; and
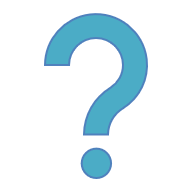
= unclear risk.

**Table SIV. Modified GRADE Approach for Umbrella Review**

|  |  | **Risk of bias** | |  |  |  |  | **Grading** | | |
| --- | --- | --- | --- | --- | --- | --- | --- | --- | --- | --- |
| **Outcomes** | **No. systematic reviews** | **Primary study** | **Systematic review (ROBIS)** | **Inconsistency** | **Indirectness** | **Imprecision** | **Publication bias**^i^ | **Initial GRADE** | **No. of downgrades** | **Final GRADE** |
| **QOL** | 17 | Serious^a^ | Serious^b^ | Not serious | Not serious | Serious^f^ | Not serious | High | 2 | Low |
| **Symptoms** | 11 | Serious^a^ | Serious^b^ | Very serious^d^ | Not serious | Not serious | Not serious | High | 3 | Very low |
| **Depression** | 10 | Serious^a^ | Serious^b^ | Not serious | Not serious | Serious^h^ | Not serious | High | 2 | Low |
| **Anxiety** | 3 | Not serious | Not serious | Not serious | Not serious | Serious^f^ | Not serious | High | 1 | Moderate |
| **Hospitalization** | 10 | Serious^a^ | Serious^b^ | Not serious | Not serious | Not serious | Not serious | High | 1 | Moderate |
| **Hospital LOS** | 1 | Not serious | Serious^c^ | Serious^e^ | Not serious | Serious^g^ | Not serious | High | 3 | Very low |
| **Mortality** | 9 | Not serious | Serious^b^ | Not serious | Not serious | Not serious | Not serious | High | 1 | Moderate |
| **Healthcare utilization (ED visits)** | 1 | Not serious | Serious^c^ | Serious^e^ | Not serious | Serious^g^ | Not serious | High | 4 | Very low |
| **Hospice referral** | 2 | Serious^a^ | Serious^b^ | Serious^e^ | Not serious | Not serious | Not serious | High | 2 | Low |
| **ACP** | 4 | Serious^a^ | Serious^b^ | Not serious | Not serious | Serious^f^ | Not serious | High | 2 | Low |
| **Satisfaction with care** | 2 | Serious^a^ | Serious^b^ | Serious^e^ | Not serious | Serious^g^ | Not serious | High | 3 | Very low |
| **Caregiver burden** | 1 | Serious^a^ | Serious^c^ | Serious^e^ | Not serious | Serious^g^ | Not serious | High | 3 | Very low |

^a^Primary studies had high risk of performance bias due to the inability to implement blinding in interventions due to the nature of interventions and detection bias for subjective outcomes. Studies had high rates of attrition leading to bias in the intervention effect estimate.

^b^Several review studies had a high risk of bias.

^c^The single review assessing this outcome had a high risk of bias.

^d^Highly inconsistent results and point estimates.

^e^Too few estimates from few studies.

^f^Large confidence intervals of primary studies and/or the overall pooled estimate.

^g^Too few events.

^h^CI crossed the no effect threshold.

^i^Publication bias was assessed by considering the comprehensiveness of the review search, search strategy, or inclusion and exclusion criteria.
